# Convergence Guarantees for Time-Inhomogeneous Uniform-Rate Discrete Diffusion Models

**DOI:** 10.3390/e28060675

**Published:** 2026-06-11

**Authors:** Yuchen Liang, Lifeng Lai, Ness Shroff, Yingbin Liang

**Affiliations:** 1 Luddy School of Informatics, Computing, and Engineering, Indiana University Indianapolis, Indianapolis, IN 46202, USA; 2 Department of Electrical and Computer Engineering, UC Davis, Davis, CA 95616, USA; 3 Department of Electrical and Computer Engineering, The Ohio State University, Columbus, OH 43210, USA

**Keywords:** discrete diffusion models, continuous-time Markov chains, time-inhomogeneity, convergence analysis

## Abstract

Discrete diffusion models have become an important class of generative models for categorical data, yet their theoretical understanding remains largely limited to time-homogeneous noise schedules. In this work, we study uniform-rate discrete diffusion models with time-inhomogeneous continuous-time Markov chain forward processes. We establish convergence guarantees for practical reverse-time samplers by directly controlling the total variation distance, avoiding the indirect route of first bounding KL divergence and then applying Pinsker’s inequality. Our analysis decomposes the sampling error into initialization, score-estimation, discretization, and early-stopping errors, and explicitly characterizes how each term depends on the accumulated noise, the local noise rate, and the smoothness of the noise schedule. Under suitable regularity conditions on the noise schedule, we further derive step-complexity guarantees that match the order of existing results for homogeneous samplers.

## 1. Introduction

Generative modeling aims to produce samples that approximate the training data distribution. Diffusion models [1,2,3] have become a leading approach, with strong performance in image, video, audio, and text generation [4,5,6,7,8]. They generate data by learning to reverse a forward noising process; for discrete data, this process is naturally modeled as a CTMC on a finite state space.

A growing line of work studies the theoretical convergence of discrete diffusion samplers. And a growing body of work suggests that for discrete data such as natural language and graphs, discrete diffusion models offer greater advantages and more flexibility than their continuous counterparts [9,10]. For uniform-rate discrete diffusion models, existing guarantees have primarily been developed for time-homogeneous CTMCs. Early work analyzed τ-leaping under total variation distance [11], while subsequent studies established convergence guarantees through score-entropy control for uniformization, exact-step, and τ-leaping-type samplers [12,13,14,15,16]. More recently, sharper analyses have been obtained for practical samplers such as the Euler method and Tweedie τ-leaping [17,18]. However, these results largely focus on homogeneous noise schedules, leaving open the question of how non-constant noise schedules affect the sampling error.

This limitation is important because time-inhomogeneous schedules are widely used in practice and can substantially affect generative performance. For continuous diffusion models, it is empirically showed in [19] that noise scheduling is a crucial design choice: the optimal schedule can depend on the task, and higher-resolution generation may benefit from schedules that place more mass on noisier regimes. These observations suggest that the noise schedule is not merely a technical detail but rather a central component controlling the trade-off between data corruption, score estimation, and reverse-time discretization. Motivated by this, we develop a convergence analysis for non-homogeneous uniform-rate discrete diffusion models. Our results quantify how the accumulated noise, the local rate $\beta_{t}$, and the smoothness of the schedule jointly determine initialization, discretization, and early-stopping errors.

## 2. Related Work

Theoretical studies of uniform-rate discrete diffusion models have so far largely considered time-homogeneous CTMCs. We have provided a summary in Table 1. An early result by [11] analyzed τ-leaping in total variation distance but required strong assumptions on the estimated reverse rates and led to suboptimal parameter dependence. Subsequent works focused on score-entropy-based guarantees for random-step samplers: ref. [12] studied a uniformization sampler on the hypercube, and ref. [16] extended the analysis to general product spaces ${\left[S\right]}^{d}$.

Other recent works analyze deterministic-step samplers. Some assume an exact per-step solver [13] or a specially designed sampler [14,20], while others study existing τ-leaping-type samplers used in practice [15,16,17]. Beyond τ-leaping, ref. [17] developed a sharp analysis for the Euler method and Tweedie τ-leaping [8], avoiding a Girsanov change-of-measure argument. Besides regular samplers, refs. [21,22] investigated possible accelerations to these vanilla samplers. Most of these results are stated in KL divergence and then converted to total variation distance, which can introduce looseness. Also, these works are focused on time-homogeneous noise schedules, which are almost never used in practice. In contrast, our work directly analyzes total variation error for time-inhomogeneous uniform-rate processes, thereby capturing the effect of non-constant schedules such as the geometric schedule used in empirical works (e.g., [8,23]).

There is a concurrent work in [24] that derives an *S*-independent upper bound on the convergence error also for the time-inhomogeneous process. Differently, their work assumes exact simulation of the continuous-time sampling process, and the step complexity when there is discretization remains unclear.

A parallel line of work studies masked, or absorbing-rate, discrete diffusion models. Convergence guarantees have been established in [15,18,20,25,26].

### Our Contributions

Our main contributions are summarized as follows:

- We establish convergence guarantees for discrete diffusion models with *time-inhomogeneous* uniform-rate generators. This extends prior analyses, which primarily focus on homogeneous noise schedules.
- We identify regularity conditions on the noise schedule under which explicit convergence rates can be obtained. Under these conditions, the resulting rates match state-of-the-art guarantees for homogeneous discrete diffusion samplers.

## 3. Preliminaries of Discrete Diffusion Samplers

In this section, we provide the background of the continuous-time discrete-space diffusion sampler.

### 3.1. Continuous-Time Forward Dynamics on Discrete State Spaces

We consider discrete data represented as a length-*d* sequence

$$x_{0}=\left(x_{0}^{1},\ldots ,x_{0}^{d}\right)\in {\left[S\right]}^{d},$$

where each coordinate $x_{0}^{i}$ takes values in a finite alphabet [S] of size *S*. Let $q_{0}^{i}$ denote the marginal probability mass function of the *i*-th token, and let $q_{0}$ denote the joint distribution of the complete data vector $x_{0}$.

The forward noising process is modeled as a time-inhomogeneous continuous-time Markov chain (CTMC) on the finite state space ${\left[S\right]}^{d}$. This process is specified by the initial law $q_{0}$ and a time-dependent transition-rate matrix $R_{t}\in R^{S^{d}\times S^{d}}$. Under the convention that $R_{t}\left(x,y\right)$ denotes the instantaneous rate of jumping from state *x* to state *y*, the infinitesimal transition probability satisfies, for sufficiently small Δt,

(1) $$q_{t+\Delta t|t}\left(y|x\right)=1\left\{y=x\right\}+R_{t}\left(x,y\right)\Delta t+o\left(\Delta t\right),\;x,y\in {\left[S\right]}^{d}.$$

Equivalently, the marginal law $q_{t}$ evolves according to the Kolmogorov forward equation

$$\frac{d}{dt}q_{t}\left(y\right)=\sum_{x\in {\left[S\right]}^{d}}q_{t}\left(x\right)R_{t}\left(x,y\right),\;y\in {\left[S\right]}^{d}.$$

For $R_{t}$ to define a valid CTMC generator, it must satisfy the standard conditions

$$R_{t}\left(x,y\right)\geq 0\;\mathrm{for}\;x\neq y,\;R_{t}\left(x,x\right)\leq 0,\;\sum_{y\in {\left[S\right]}^{d}}R_{t}\left(x,y\right)=0.$$

Directly specifying a generator over the full space ${\left[S\right]}^{d}$ is generally infeasible when either *S* or *d* is large. A common simplification is therefore to impose coordinate-wise independent corruption so that only one token changes at any infinitesimal transition [8,11]. In this case, for x≠y, the generator takes the form [11] (Prop. 3)

(2) $$R_{t}\left(x,y\right)=\left\{\begin{array}{cc} R_{t}^{\mathrm{tok}}\left(x^{i},y^{i}\right),\; & \mathrm{if}\;\mathrm{Ham}\left(x,y\right)=1\;\mathrm{and}\;x^{i}\neq y^{i},\\ 0,\; & \mathrm{if}\;\mathrm{Ham}\left(x,y\right)>1, \end{array}\right.$$

where $R_{t}^{\mathrm{tok}}\in R^{S\times S}$ is the token-level transition-rate matrix and $\mathrm{Ham}\left(x,y\right)$ denotes the Hamming distance between *x* and *y*. The diagonal entries of $R_{t}$ are then determined by the zero-row-sum constraint.

Following [11], we parameterize the token-level generator as

$$R_{t}^{\mathrm{tok}}=\beta_{t}R_{\mathrm{base}},$$

where $\beta_{t}>0$ is a noise schedule and $R_{\mathrm{base}}$ is a fixed base generator. Prior work often studies the homogeneous case $\beta_{t}\equiv 1$; see, for example, refs. [13,16]. We define the accumulated noise level by

$${\bar{\beta }}_{t}:=\int_{0}^{t}\beta_{s}\;ds<\infty .$$

Since $\beta_{t}>0$, the map $t\mapsto {\bar{\beta }}_{t}$ is increasing. We denote the inverse of ${\bar{\beta }}_{t}$ as ${\bar{\beta }}^{-1}$, so that

$${\bar{\beta }}^{-1}\left({\bar{\beta }}_{t}\right)=t.$$

Throughout, we assume that $\beta_{t}$ is smooth on [0,T].

The coordinate-wise construction yields a tractable closed-form transition kernel. In particular, the token-level transition matrix from time 0 to time *t* is

$$Q_{t|0}^{\mathrm{tok}}=\exp\;\left({\bar{\beta }}_{t}R_{\mathrm{base}}\right),$$

and the full transition kernel factorizes across coordinates as

$$q_{t|0}\left(x_{t}|x_{0}\right)=\prod_{i=1}^{d}Q_{t|0}^{\mathrm{tok}}\left(x_{0}^{i},x_{t}^{i}\right).$$

This closed form is especially useful for training discrete diffusion models.

In this work, we focus on the commonly used uniform-rate discrete diffusion model, with

$$R_{\mathrm{base}}:=\frac{1}{S}1_{S}1_{S}^{⊺}-I_{S},$$

which appears in several prior works [3,8,11]. For this choice, each token is driven toward the uniform distribution over [S]. Moreover, the diagonal entry of the full-state generator is

$$R_{t}\left(x,x\right)=-\sum_{y:y\neq x}R_{t}\left(x,y\right)=-\beta_{t}\frac{S-1}{S}d,\;x\in {\left[S\right]}^{d}.$$

With such an $R_{t}$, as *T* becomes large, the terminal distribution induced by this forward process approaches the uniform distribution on ${\left[S\right]}^{d}$.

### 3.2. Reverse Dynamics and Discrete-Time Sampling

The CTMC forward process induces a time-reversed CTMC whose marginals coincide with those of the forward chain run backward in time [27]. More precisely, by [11] (Prop. 1), the reverse process starts from

$${\;\overset{\leftarrow }{\;q\;}\;}_{0}:=q_{T}$$

and evolves with transition-rate matrix ${\;\overset{\leftarrow }{\;R\;}\;}_{t}$ given, for x≠y, by

(3) $${\;\overset{\leftarrow }{\;R\;}\;}_{t}\left(x,y\right)=R_{T-t}\left(y,x\right)\frac{q_{T-t}\left(y\right)}{q_{T-t}\left(x\right)},\;x,y\in {\left[S\right]}^{d},$$

with diagonal entries determined by

$${\;\overset{\leftarrow }{\;R\;}\;}_{t}\left(x,x\right)=-\sum_{y:y\neq x}{\;\overset{\leftarrow }{\;R\;}\;}_{t}\left(x,y\right).$$

With this setup, the marginal distribution of this reverse chain satisfies

$${\;\overset{\leftarrow }{\;q\;}\;}_{t}=q_{T-t}.$$

Under the coordinate-wise forward generator in (2), the reverse generator inherits the same sparsity pattern: if $\mathrm{Ham}\left(x,y\right)\geq 2$, then

$${\;\overset{\leftarrow }{\;R\;}\;}_{t}\left(x,y\right)=0.$$

Thus, both the forward and reverse processes only allow infinitesimal transitions that modify a single token. In practice, the reverse process is stopped at time T−δ for a small δ>0. This early-stopping convention avoids the numerical instability that may arise as the forward time approaches $0^{+}$, where the corresponding score ratios can become irregular (as shown in [17] (Lemma 2)).

The exact reverse CTMC is not directly implementable, and the sampling procedure therefore introduces several approximations. Let $p_{t}$ denote the marginal distribution of the practical sampler at reverse time t∈[0,T−δ]. First, the exact initial law $q_{T}$ is typically unavailable. Since the forward process with the uniformizing base generator converges to the uniform distribution, the sampler is initialized as

$$p_{0}:=\mathrm{Uniform}\left({\left[S\right]}^{d}\right).$$

Second, the likelihood ratio appearing in (3) is unknown. For reverse time *t*, the required ratio $\frac{q_{T-t}\left(y\right)}{q_{T-t}\left(x\right)}$ is approximated by a learned concrete score function

$$s_{T-t}\left(y,x\right)\approx \frac{q_{T-t}\left(y\right)}{q_{T-t}\left(x\right)}.$$

Consequently, on a discrete sampling grid, the reverse generator is replaced by an estimated generator.

Beyond estimation error, we also account for the error introduced by approximate sampling procedures. Let

$$0=t_{0}<t_{1}<\cdots <t_{N}=T-\delta$$

be the reverse-time discretization grid. At grid point $t_{k}$, the off-diagonal entries of the estimated reverse generator are

(4) $${\hat{R}}_{t_{k}}\left(x,y\right):=R_{T-t_{k}}\left(y,x\right)s_{T-t_{k}}\left(y,x\right),\;x\neq y,$$

with

$${\hat{R}}_{t_{k}}\left(x,x\right)=-\sum_{y:y\neq x}{\hat{R}}_{t_{k}}\left(x,y\right).$$

This approximation replaces the exact reverse rate in (3) by a score-based estimate.

Following [8,23], one example is to use the Euler method that freezes the estimated generator over each interval $\left[t_{k},t_{k+1}\right)$. Since the generator only permits single-token transitions, the update can be written in a token-wise form. Given the current state $x_{t_{k}}$, define, for each coordinate i∈[d] and candidate token a∈[S] with $a\neq x_{t_{k}}^{i}$,

(5) $${\hat{R}}_{k}^{i}\left(x_{t_{k}}^{i},a\right):={\hat{R}}_{t_{k}}\left(x_{t_{k}},x_{t_{k}}^{-i}{⊕}_{i}a\right),$$

where $x_{t_{k}}^{-i}{⊕}_{i}a$ denotes the vector obtained from $x_{t_{k}}$ by replacing its *i*-th token with *a*. The corresponding diagonal token rate is

$${\hat{R}}_{k}^{i}\left(x_{t_{k}}^{i},x_{t_{k}}^{i}\right):=-\sum_{a:a\neq x_{t_{k}}^{i}}{\hat{R}}_{k}^{i}\left(x_{t_{k}}^{i},a\right).$$

The Euler transition for the *i*-th token is then

(6) $$P\left(x_{t_{k+1}}^{i}=a|x_{t_{k}}\right)=\left\{\begin{array}{cc} \left(t_{k+1}-t_{k}\right){\hat{R}}_{k}^{i}\left(x_{t_{k}}^{i},a\right),\; & a\neq x_{t_{k}}^{i},\\ 1+\left(t_{k+1}-t_{k}\right){\hat{R}}_{k}^{i}\left(x_{t_{k}}^{i},x_{t_{k}}^{i}\right),\; & a=x_{t_{k}}^{i}. \end{array}\right.$$

Equivalently, each token either remains unchanged or jumps to another symbol according to the local estimated reverse rates. The step size is typically small, so that the probabilities in (6) remain nonnegative. The final output of the sampler is the state at reverse time T−δ, whose law is intended to approximate the early-stopped data distribution $q_{\delta }$.

### 3.3. Notations

Let $x^{i}\left(1\leq i\leq d\right)$ denote the *i*-th element of a vector $x\in {\left[S\right]}^{d}$ and $x^{-i}\in {\left[S\right]}^{d-1}$ denote the *i*-th element removed. Define $\mathrm{Ham}\left(x,y\right)$ as the Hamming distance between two vectors *x* and *y*. For a positive integer *n*, [n]:={1,…,n}. Write $1_{S}$ as a vector of length *S* that contains all 1’s, and $I_{S}$ as an identity matrix of size S×S.

## 4. Convergence Under General Non-Homogeneous Noise Schedule

In this section, we establish improved upper error bounds and convergence rates for the uniform-rate matrix. Following the approach of [18], our results are stated directly in terms of total variation distance rather than first deriving a KL-divergence bound and then applying Pinsker’s inequality. Our analysis relies on the following estimation-error condition.

**Assumption** **1.** *The score estimation error satisfies that $L_{TV}\left(s\right)\leq \sqrt{\varepsilon_{\mathrm{TV}}}$, where*

$$L_{TV}\left(s\right):=\sum_{k=0}^{N-1}\left(t_{k+1}-t_{k}\right)E_{x_{k}∼q_{T-t_{k}}}\left[\sum_{y:y\neq x_{k}}R_{T-t_{k}}\left(y,x_{k}\right)\left|s_{T-t_{k}}\left(y,x_{k}\right)-\frac{q_{T-t_{k}}\left(y\right)}{q_{T-t_{k}}\left(x_{k}\right)}\right|\right].$$

Assumption 1 requires the learned score to be accurate at $L_{1}$ distance, measured as the expected sum of absolute differences between the exact and estimated rate matrices. This condition differs from the commonly used score-entropy loss [16,17] but is particularly convenient for our direct total-variation-based analysis. For masked diffusion models, ref. [18] shows that $L_{TV}$ can be upper-bounded by the score-entropy loss. We have provided a similar justification in Appendix A.

With this condition in place, the following theorem provides an upper bound on the sampling error in total variation distance.

**Theorem** **1.** *Suppose that Assumption 1 holds. As long as $t_{k+1}-t_{k}$ vanishes at small ε and that each Euler step is valid, we have*

$$\begin{array}{cc} \mathrm{TV}\left(q_{0},p_{T-\delta }\right)≲\min\{d, & \sqrt{dS}\}e^{-{\bar{\beta }}_{T}}+\sqrt{\varepsilon_{\mathrm{TV}}}\\ & +dS\sum_{k=0}^{N-1}\max\left\{1,{\bar{\beta }}_{T-t_{k+1}}^{-2}\right\}\left(\right|\beta_{T-t_{k}}^{\prime }\left|+d\beta_{T-t_{k}}^{2}\right){\left(t_{k+1}-t_{k}\right)}^{2}+d{\bar{\beta }}_{\delta }. \end{array}$$

Theorem 1 decomposes the total sampling error into four distinct contributions. The first term, $\min\left\{d,\sqrt{dS}\right\}e^{-{\bar{\beta }}_{T}}$, corresponds to the initialization error, which quantifies the discrepancy between the true terminal distribution $q_{T}$ and the uniform distribution used to initialize the sampler. This error decays exponentially fast with the accumulated noise level ${\bar{\beta }}_{T}$. The second term, $\sqrt{\varepsilon_{\mathrm{TV}}}$, captures the error due to imperfect score estimation under Assumption 1. The third term represents the discretization error incurred by replacing the continuous-time reverse CTMC with a discrete-time approximation on the grid ${\left\{t_{k}\right\}}_{k=0}^{N}$. Finally, the last term, $d{\bar{\beta }}_{\delta }$, is the early-stopping error, which arises because the reverse process is terminated at time T−δ rather than being run all the way to the data distribution.

**Remark** **1** (On the time steps $t_{k+1}-t_{k}$)**.** *In order to guarantee the validity of the Euler steps, it is necessary to have that*

$$|{\hat{R}}_{k}^{i}\left(x^{i},x^{i}\right)|=\sum_{a:a\neq x^{i}}{\hat{R}}_{k}^{i}\left(x^{i},a\right)<1.$$

*Assuming that the score functions are accurately estimated, from Lemma A2 we have $|{\hat{R}}_{k}^{i}\left(x^{i},x^{i}\right)|≲S$, necessarily yielding $t_{k+1}-t_{k}=o\left(1/S\right)$. Note that this condition is satisfied for the step size in Theorem 2.*

**Remark** **2** (On the early-stopping parameter δ)**.** *For time-homogeneous models, to achieve $O(\sqrt{\varepsilon })$ error, δ can be easily set to be $\sqrt{\varepsilon }/d$ (see Corollary 1). For non-homogeneous schedules, however, such a dependence on d becomes implicit, and one should set δ such that ${\bar{\beta }}^{-1}\left(\sqrt{\varepsilon }/d\right)$, which depends on the choice of the actual schedule.*

For comparison, we provide the following corollary for the special case of a time-homogeneous CTMC, which is immediate upon plugging in $\beta_{t}\equiv 1$.

**Corollary** **1.** *Under the setting of Theorem 1, for time-homogeneous CTMC, i.e., when $\beta_{t}\equiv 1$, we have*

$$\begin{array}{cc} \mathrm{TV}\left(q_{0},p_{T-\delta }\right)≲\min\left\{d,\sqrt{dS}\right\}e^{-T}+ & \sqrt{\varepsilon_{\mathrm{TV}}}\\ & +d^{2}S\sum_{k=0}^{N-1}\max\left\{1,{\left(T-t_{k+1}\right)}^{-2}\right\}{\left(t_{k+1}-t_{k}\right)}^{2}+d\delta . \end{array}$$

Compared with the time-homogeneous CTMC setting, our error bound in Theorem 1 exhibits two key differences. First, the initialization and early-stopping errors are no longer determined solely by the diffusion times *T* and δ; they also depend on the prescribed noise schedule through the accumulated noise levels ${\bar{\beta }}_{T}$ and ${\bar{\beta }}_{\delta }$. Second, the discretization error is not controlled only by the selected time steps $\left(t_{k+1}-t_{k}\right)$. Instead, it additionally depends on the smoothness of the noise schedule through $|\beta_{T-t_{k}}^{\prime }|$ and the magnitude of the transition rates through $\beta_{T-t_{k}}^{2}$, which is, effectively speaking, the size of each discretization interval as measured by the amount of noise injected over that interval. We have provided a proof sketch in Section 5.

To further determine the number of steps to reach $O(\sqrt{\varepsilon })$ error, we consider those $\beta_{t}$’s that satisfy the following regularity condition.

**Assumption** **2** (Slow-varying noise)**.** *Suppose that noise schedule* $\beta_{t}$ *satisfies the following regularity conditions:*

*1.*  *The schedule is uniformly bounded away from zero on the relevant low-noise interval, namely,*
$$\beta^{*}:=\min_{z\in [\delta ,{\bar{\beta }}^{-1}\left(1\right)]}\beta_{z}≳1.$$
*2.*  *The schedule is asymptotically constant (ignoring logarithms):*
$$\beta_{t},\;\left|\beta_{t}^{\prime }\right|=\tilde{O}\left(1\right),\;\forall t\in \left[\delta ,T\right].$$
*3.*  *The inverse accumulated-noise map grows at most polynomially:*
$${\bar{\beta }}^{-1}\left(x\right)=\mathrm{poly}\left(x\right).$$

On a high level, Assumption 2 assumes that the noise schedule is slow-varying. This assumption is used only to determine a suitable choice of the number of sampling steps. In the time-homogeneous CTMC setting, where $\beta_{t}\equiv 1$, we have $\beta^{*}=1$ and $\beta_{t}^{\prime }\equiv 0$. Therefore, the homogeneous case automatically satisfies Assumption 2. Another example of noise schedule that satisfies Assumption 2 is the polynomial schedule where ${\bar{\beta }}_{t}=t^{p}$, where p>0. With this choice, $\beta_{t}=pt^{p-1}$ and $\beta^{*}=p\delta^{p-1}$ if p≥1 and *p* otherwise. ${\bar{\beta }}^{-1}\left(x\right)=x^{1/p}=\mathrm{poly}\left(x\right)$. Further, for t∈[δ,T], $\beta_{t}=pt^{p-1}\leq pT^{p-1}$ and $|\beta_{t}^{\prime }\left|\leq \right|p\left(p-1\right)T^{p-2}|$, for which we note that $T=\mathrm{poly}(\log\left(d/\sqrt{\varepsilon }\right))$ is asymptotically a constant. In general, both the initial $\beta_{0}$ and the growth of $\beta_{t}$ w.r.t. *t* need to be mild to satisfy Assumption 2.

Beyond polynomial schedule, the exponential (also known as geometric) noise schedule does not satisfy this assumption. This will be our main focus in Section 6.

The first condition in Assumption 2 imposes a constant lower bound on $\beta_{t}$ over the low-noise regime. Intuitively, this prevents the noise schedule from becoming too small near the data distribution, ensuring that the score function does not blow up over the entire reverse process. The second condition controls the cumulative magnitude and variation of the schedule along the discretization grid. This requirement ensures that the transition rates do not depend on the system parameters, which is a common practice, so that the discrete-time approximation remains stable and reliable. Finally, the third condition requires the inverse accumulated-noise map ${\bar{\beta }}^{-1}$ to grow at most polynomially. Equivalently, this guarantees that one can reach a sufficiently large terminal accumulated noise level ${\bar{\beta }}_{T}$ within a polynomial diffusion time, thereby ensuring that enough noise is injected by the terminal time.

With Assumption 2, the following theorem characterizes the number of sampling steps required to achieve $O(\sqrt{\varepsilon })$ error in the time-inhomogeneous setting.

**Theorem** **2.** *Under the setting of Theorem 1, suppose further that Assumption 2 holds. Let $t_{k+1}-t_{k}=\kappa \min\left\{1,{\bar{\beta }}_{T-t_{k}}\right\}$. Then, in order to achieve $O(\sqrt{\varepsilon })$ TV-error, one can choose $T={\bar{\beta }}^{-1}\left(\log\left(d/\sqrt{\varepsilon }\right)\right)$, $\kappa ≍\sqrt{\varepsilon }/\left(d^{2}S\right)$, and thus $N=\tilde{O}\left(d^{2}S/\sqrt{\varepsilon }\right)$.*

The proof of Theorem 2 is in Appendix C. This theorem shows that under the stated regularity conditions on the noise schedule, the time-inhomogeneous sampler requires the same order of sampling steps as in the time-homogeneous setting; see, e.g., refs. [17,18]. The main difference is that in the inhomogeneous case, both the discretization step sizes and the terminal diffusion time must be chosen according to the prescribed noise schedule.

## 5. Proof Sketch of Theorem 1

We now provide a proof sketch of Theorem 1. The full proof is in Appendix B.

**Proof** **sketch of Theorem 1.** We analyze the Euler sampler through the truncated τ-leaping sampler, which is asymptotically equivalent to Euler by [17] (Lemma 8). The key property is that the estimated rate is piecewise constant on each interval $\left[t_{k},t_{k+1}\right)$:

$${\hat{R}}_{t}\left(x_{t_{k}},\cdot \right)={\hat{R}}_{t_{k}}\left(x_{t_{k}},\cdot \right),\;t\in \left[t_{k},t_{k+1}\right).$$

Let ${\;\overset{\leftarrow }{\;q\;}\;}_{t}=q_{T-t}$ denote the exact reverse marginal. By the TV perturbation bound of [18] (Theorem 1),

$$\mathrm{TV}\left({\;\overset{\leftarrow }{\;q\;}\;}_{T-\delta },p_{T-\delta }\right)\leq \mathrm{TV}\left({\;\overset{\leftarrow }{\;q\;}\;}_{0},p_{0}\right)+\sum_{k=0}^{N-1}\int_{t_{k}}^{t_{k+1}}E_{x_{t}∼{\;\overset{\leftarrow }{\;q\;}\;}_{t}}\sum_{y:y\neq x_{t}}\left|{\hat{R}}_{t}\left(x_{t},y\right)-{\;\overset{\leftarrow }{\;R\;}\;}_{t}\left(x_{t},y\right)\right|dt.$$

Writing

$$h_{t}\left(x\right):=\sum_{y:y\neq x}\left|{\hat{R}}_{t}\left(x,y\right)-{\;\overset{\leftarrow }{\;R\;}\;}_{t}\left(x,y\right)\right|,$$

and adding and subtracting $h_{t_{k}}\left(x_{t_{k}}\right)$, the error decomposes into initialization, score-estimation, and discretization terms. By Assumption 1, the score-estimation term is bounded by

$$\sum_{k=0}^{N-1}\left(t_{k+1}-t_{k}\right)E_{x_{t_{k}}∼{\;\overset{\leftarrow }{\;q\;}\;}_{t_{k}}}h_{t_{k}}\left(x_{t_{k}}\right)\leq \sqrt{\varepsilon_{\mathrm{TV}}}.$$

The initialization error is controlled by the convergence of the forward chain to the uniform distribution. Since ${\;\overset{\leftarrow }{\;q\;}\;}_{0}=q_{T}$ and $p_{0}=\mathrm{Uniform}\left({\left[S\right]}^{d}\right)$, we have

$$\mathrm{TV}\left({\;\overset{\leftarrow }{\;q\;}\;}_{0},p_{0}\right)=\mathrm{TV}\left(q_{T},p_{0}\right)≲\min\left\{d,\sqrt{dS}\right\}e^{-{\bar{\beta }}_{T}}.$$

It remains to bound the discretization error. The part caused by the change from $x_{t_{k}}$ to $x_{t}$ is lower-order for sufficiently small step size, since the total outgoing reverse rate is of order $d\beta_{T-t}$. The dominant contribution comes from the time variation of the reverse rate. Using the piecewise-constant property of ${\hat{R}}_{t}$,

$$E_{x_{t_{k}}∼{\;\overset{\leftarrow }{\;q\;}\;}_{t_{k}}}\left[h_{t}\left(x_{t_{k}}\right)-h_{t_{k}}\left(x_{t_{k}}\right)\right]≲E_{x_{t_{k}}∼{\;\overset{\leftarrow }{\;q\;}\;}_{t_{k}}}\sum_{y:y\neq x_{t_{k}}}\left|{\;\overset{\leftarrow }{\;R\;}\;}_{t}\left(x_{t_{k}},y\right)-{\;\overset{\leftarrow }{\;R\;}\;}_{t_{k}}\left(x_{t_{k}},y\right)\right|.$$

By the reverse-rate formula in (3) and its coordinate-wise structure, only states satisfying $\mathrm{Ham}\left(x,y\right)=1$ contribute. The difference is then split into the change in the likelihood ratio and the change in the rate matrix. The likelihood-ratio term is bounded using the non-homogeneous score-ratio estimate

$$\frac{q_{s}\left(y\right)}{q_{s}\left(x\right)}≲S\max\left\{1,{\bar{\beta }}_{s}^{-1}\right\},\;\forall \left(x,y\right):\mathrm{Ham}\left(x,y\right)=1,$$

together with the corresponding likelihood-ratio difference bound. The rate-matrix term follows from smoothness of $\beta_{t}$:

$$|R_{T-t_{k}}\left(y,x\right)-R_{T-t}\left(y,x\right)|≲\frac{1}{S}\left|\beta_{T-t_{k}}^{\prime }\right|\left(t-t_{k}\right).$$

Combining these bounds yields

$$D_{\mathrm{disc}}≲dS\sum_{k=0}^{N-1}\max\left\{1,{\bar{\beta }}_{T-t_{k+1}}^{-2}\right\}\left(|\beta_{T-t_{k}}^{\prime }|+d\beta_{T-t_{k}}^{2}\right){\left(t_{k+1}-t_{k}\right)}^{2},$$

where $D_{\mathrm{disc}}$ represents the discretization error.

Finally, because the reverse process is stopped at T−δ, its target marginal is $q_{\delta }$ rather than $q_{0}$. The early-stopping error satisfies

$$\mathrm{TV}\left(q_{0},q_{\delta }\right)≲d{\bar{\beta }}_{\delta }.$$

Using the triangle inequality,

$$\mathrm{TV}\left(q_{0},p_{T-\delta }\right)\leq \mathrm{TV}\left(q_{0},q_{\delta }\right)+\mathrm{TV}\left({\;\overset{\leftarrow }{\;q\;}\;}_{T-\delta },p_{T-\delta }\right),$$

and combining the four bounds gives the desired result. □

## 6. Geometric Noise Schedule

While the geometric schedule [8] does not directly satisfy Assumption 2, the preceding time-inhomogeneous framework enables us to analyze such a noise schedule. Different from the common scenario where *T* diverges, in practice one typically sets T=1 without diverging with vanishing ε. This way, the geometric noise schedule is defined as follows.

(7) $${\bar{\beta }}_{t}:=\beta_{\min}^{1-t}\beta_{\max}^{t},\;\beta_{t}=\beta_{\min}^{1-t}\beta_{\max}^{t}\log\left(\beta_{\max}/\beta_{\min}\right)={\bar{\beta }}_{t}\log\left(\beta_{\max}/\beta_{\min}\right),\;t\in \left[0,1\right],$$

where $0<\beta_{\min}<1<\beta_{\max}$ are prescribed constants. The default choice in [8] is $\beta_{\min}=10^{-4}$ and $\beta_{\max}=20$. Here, both ${\bar{\beta }}_{t}$ and $\beta_{t}$ are increasing functions on [0,1]. Moreover, differentiating $\beta_{t}$ gives

$$\beta_{t}^{\prime }=\beta_{\min}^{1-t}\beta_{\max}^{t}\log^{2}\left(\beta_{\max}/\beta_{\min}\right)=\beta_{t}\log\left(\beta_{\max}/\beta_{\min}\right).$$

We first follow ref. [8] and use a constant step size κ, so that the total number of discretization steps is $N=\kappa^{-1}$. The following theorem specializes our general time-inhomogeneous result to the geometric noise schedule.

**Theorem** **3.** *Suppose that Assumption 1 holds. With the geometric noise schedule defined in* (7), *choosing $t_{k+1}-t_{k}=\kappa$ where $t_{0}=0$ and $t_{N}=1-\delta$, we have*

$$\begin{array}{cc} \lim_{\delta \to 0^{+}}\mathrm{TV}\left(q_{0},p_{1-\delta }\right)≲\min & \left\{d,\sqrt{dS}\right\}e^{-\beta_{\max}}+\sqrt{\varepsilon_{\mathrm{TV}}}\\ & +\kappa d^{2}S\log\left(\beta_{\max}/\beta_{\min}\right)\left({\left(\beta_{\min}^{-1}-1\right)}^{2}+{\left(\beta_{\max}-1\right)}^{2}\right)+d\beta_{\min}. \end{array}$$

*Thus, in order to achieve $O(\sqrt{\varepsilon })$ TV error, we can choose*

$$\beta_{\min}≍\frac{\sqrt{\varepsilon }}{d},\;\beta_{\max}≍\log\frac{\min\left\{d,\sqrt{dS}\right\}}{\sqrt{\varepsilon }},\;\;\mathrm{and}\;\;\kappa ≍\frac{\varepsilon^{3/2}}{d^{4}S\log\left(d/\varepsilon \right)},$$

*we have $N=\tilde{O}\left(d^{4}S/\varepsilon^{3/2}\right)$.*

Theorem 3 highlights an interesting regime in which a finite error upper bound can be obtained even as $\delta \to 0^{+}$. This contrasts with prior bounds whose dependence on $\log\delta^{-1}$ diverges in the vanishing early-stopping limit [15,16,17,18]. While the initialization, score-estimation, and early-stopping terms retain the same interpretation as in the general time-inhomogeneous result, the discretization error admits an explicit expression under the geometric noise schedule. In particular, up to logarithmic factors, this term scales quadratically with the prescribed schedule parameters $\beta_{\min}^{-1}$ and $\beta_{\max}$.

Our result illustrates the trade-off governed by the schedule parameters $\beta_{\min}$ and $\beta_{\max}$, when the step sizes are constant, as is typical in many empirical studies [8,23]. A smaller $\beta_{\min}$ reduces the early-stopping error but increases the accumulated discretization error. Similarly, a larger $\beta_{\max}$ decreases the initialization error while again amplifying the discretization error.

Note that the choice of the sampling steps is not optimized here (i.e., constant) and therefore may lead to a suboptimal overall step complexity. As in Theorem 2, we can similarly employ a constant-then-decreasing step size towards the end to obtain an improved convergence result, given as follows.

**Theorem** **4.** *With the same setting as in Theorem 3, but choosing $t_{k+1}-t_{k}=\kappa \min\left\{1,\beta_{\min}^{t_{k}}\beta_{\max}^{1-t_{k}}\right\}$, we have*

$$\begin{array}{cc} \lim_{\delta \to 0^{+}}\mathrm{TV}\left(q_{0},p_{1-\delta }\right)≲\min\left\{d,\sqrt{dS}\right\} & e^{-\beta_{\max}}+\sqrt{\varepsilon_{\mathrm{TV}}}\\ & +\kappa d^{2}S\log\left(\beta_{\max}/\beta_{\min}\right)\left({\left(\beta_{\max}-1\right)}^{2}+1\right)+d\beta_{\min}. \end{array}$$

*Meanwhile, the number of steps satisfies that*

$$N≲\frac{1}{\kappa }\left(1+\frac{\beta_{\min}^{-1}-1}{\log(\beta_{\max}/\beta_{\min})}\right).$$

*Thus, choosing*

$$\beta_{\min}≍\frac{\sqrt{\varepsilon }}{d},\;\beta_{\max}≍\log\frac{\min\left\{d,\sqrt{dS}\right\}}{\sqrt{\varepsilon }},\;\;\mathrm{and}\;\;\kappa ≍\frac{\sqrt{\varepsilon }}{d^{2}S\log\left(d/\varepsilon \right)},$$

*we have $N=\tilde{O}\left(d^{3}S/\varepsilon \right)$.*

Compared to Theorem 3, Theorem 4 improves the step complexity by a factor of order $O(d/\sqrt{\varepsilon })$. The key observation is that under the geometric noise schedule, the effective noise level decays exponentially along the reverse-time trajectory. The exponential-then-constant step size adapts to this behavior: it uses relatively large constant steps in the high-noise regime, where the reverse dynamics are more stable, and automatically takes smaller steps near the low-noise regime, where the discretization error is most sensitive. This refinement removes the unfavorable dependence on $\beta_{\min}^{-2}$ that appears under a constant step size while increasing the number of steps only in the region where finer discretization is necessary. As a result, the adaptive scheme achieves a sharper overall sampling complexity.

As a future direction, it might be interesting to investigate why using such a geometric noise schedule would yield worsened results than using polynomial ones.

## 7. Conclusions

In conclusion, this work provides a theoretical foundation for discrete diffusion models driven by time-inhomogeneous uniform-rate generators. By moving beyond the homogeneous noise schedules considered in much of the prior literature, our analysis broadens the class of discrete diffusion processes for which convergence can be rigorously understood. We further identify regularity conditions on the noise schedule that enable explicit convergence-rate estimates. Under these conditions, the resulting guarantees match state-of-the-art rates known for homogeneous discrete diffusion samplers, demonstrating that carefully controlled time-inhomogeneous schedules can retain the same theoretical efficiency while offering greater modeling flexibility.

## Figures and Tables

**Table 1:** Summary of results for regular uniform-rate discrete diffusion samplers in terms of the number of steps needed to achieve $\sqrt{\varepsilon }$-accuracy in $\mathrm{TV}(q_{\delta },p_{T-\delta })$. Only the best result for each sampler is shown. All log-dependencies are omitted. In the table, *d* is the dimension, *S* is the vocabulary size, and *M* is an upper bound of the score estimates. In this work, we provide the first analysis under non-homogeneous noise schedule and $L_{1}$ estimation error (i.e., in total-variation metric), having a convergence rate comparable to the state-of-the-art results.

Sampler	Assumption	Time-Homo-Geneous?	Results: Num of Steps	Reference
Kolmogorov	Score entropy, bounded score	Yes	$O\left(\frac{\sqrt{dM}S}{\sqrt{\varepsilon }}\right)$	[13]
DMPM	Score entropy	Yes	$O\left(\frac{dS^{2}}{\varepsilon^{2}}\right)$	[14,20]
τ-leaping	$L_{\infty }$ error	Yes	$O\left(\frac{d^{4}S^{4}}{\sqrt{\varepsilon }}\right)$	[11]
τ-leaping	Score entropy, bounded score	Yes	$O\left(\frac{d^{2}S^{2}}{\varepsilon }\right)$	[16,17]
τ-leaping	Score entropy	Yes	$O\left(\frac{d}{\varepsilon }\right)$	[15]
Euler method, Tweedie τ-leaping	Score entropy, bounded score	Yes	$O\left(\frac{d^{2}S}{\varepsilon }\right)$	[17]
Euler method, Tweedie τ-leaping	$L_{1}$ error, slow-varying noise	No	$O\left(\frac{d^{2}S}{\sqrt{\varepsilon }}\right)$	Theorem 2

## Data Availability

Data sharing is not applicable. No new data were created or analyzed in this study.

## Appendix A. Justification of Assumption 1

The following proposition links the TV score-estimation error in Assumption 1 with the commonly used score-entropy loss [8].

**Proposition** **A1.**
*Define the score entropy loss at time t as*

$$L_{SE}\left(s;t\right)=E_{x_{t}∼q_{t}}\sum_{y:y\neq x_{t}}R_{t}\left(y,x_{t}\right)\cdot \left(s_{t}\left(y,x\right)-\frac{q_{t}\left(y\right)}{q_{t}\left(x\right)}-\frac{q_{t}\left(y\right)}{q_{t}\left(x\right)}\log\frac{s_{t}\left(y,x\right)}{q_{t}\left(y\right)/q_{t}\left(x\right)}\right).$$

*If ${\hat{R}}_{t}\left(x,y\right)-{\;\overset{\leftarrow }{\;R\;}\;}_{t}\left(x,y\right)=o\left(1\right),\;\forall t,x,y$, then we have*

$$L_{TV}\left(s;T-t\right)≲\sqrt{d(S-1)}\max\left\{1,{\bar{\beta }}_{T-t}^{-1}\right\}\cdot \sqrt{L_{SE}\left(s;T-t\right)}.$$

**Proof** **Proposition A1.** Recall that ${\hat{R}}_{t_{k}}\left(x_{k},y\right)=R_{T-t_{k}}\left(y,x_{k}\right)s_{T-t_{k}}\left(y,x_{k}\right)$. From the definition, the TV loss at time *t* satisfies that

$$\begin{array}{cc} L_{TV}^{2}\left(s;T-t_{k}\right) & ={\left(E_{x_{k}∼{\;\overset{\leftarrow }{\;q\;}\;}_{t_{k}}}\left[\sum_{y:y\neq x_{k}}\left|{\hat{R}}_{t_{k}}\left(x_{k},y\right)-{\;\overset{\leftarrow }{\;R\;}\;}_{t_{k}}\left(x_{k},y\right)\right|\right]\right)}^{2}\\ \; & \leq E_{x_{k}∼{\;\overset{\leftarrow }{\;q\;}\;}_{t_{k}}}{\left(\sum_{y:y\neq x_{k}}\left|{\hat{R}}_{t_{k}}\left(x_{k},y\right)-{\;\overset{\leftarrow }{\;R\;}\;}_{t_{k}}\left(x_{k},y\right)\right|\right)}^{2}\\ \; & =d^{2}{\left(S-1\right)}^{2}E_{x_{k}∼{\;\overset{\leftarrow }{\;q\;}\;}_{t_{k}}}{\left(\frac{1}{d(S-1)}\sum_{y:y\neq x_{k}}\left|{\hat{R}}_{t_{k}}\left(x_{k},y\right)-{\;\overset{\leftarrow }{\;R\;}\;}_{t_{k}}\left(x_{k},y\right)\right|\right)}^{2}\\ \; & \leq E_{x_{k}∼{\;\overset{\leftarrow }{\;q\;}\;}_{t_{k}}}d^{2}{\left(S-1\right)}^{2}\frac{1}{d(S-1)}\sum_{y:y\neq x_{k}}{\left({\hat{R}}_{t_{k}}\left(x_{k},y\right)-{\;\overset{\leftarrow }{\;R\;}\;}_{t_{k}}\left(x_{k},y\right)\right)}^{2}\\ \; & \leq d\left(S-1\right)E_{x_{k}∼{\;\overset{\leftarrow }{\;q\;}\;}_{t_{k}}}\sum_{y:y\neq x_{k}}{\left({\hat{R}}_{t_{k}}\left(x_{k},y\right)-{\;\overset{\leftarrow }{\;R\;}\;}_{t_{k}}\left(x_{k},y\right)\right)}^{2} \end{array}$$

where both inequalities are due to Jensen’s inequality. On the other hand, the score-entropy loss at time *t* satisfies that

$$\begin{array}{cc} L_{SE}\left(s;T-t_{k}\right) & =E_{x_{t}∼{\;\overset{\leftarrow }{\;q\;}\;}_{t_{k}}}\sum_{y:y\neq x_{k}}{\hat{R}}_{t_{k}}\left(x_{k},y\right)-{\;\overset{\leftarrow }{\;R\;}\;}_{t_{k}}\left(x_{k},y\right)-{\;\overset{\leftarrow }{\;R\;}\;}_{t_{k}}\left(x_{k},y\right)\log\frac{{\hat{R}}_{t_{k}}\left(x_{k},y\right)}{{\;\overset{\leftarrow }{\;R\;}\;}_{t_{k}}\left(x_{k},y\right)}\\ \; & \overset{\left(i\right)}{=}E_{x_{t}∼{\;\overset{\leftarrow }{\;q\;}\;}_{t_{k}}}\sum_{y:y\neq x_{k}}\frac{{\left({\hat{R}}_{t_{k}}\left(x_{k},y\right)-{\;\overset{\leftarrow }{\;R\;}\;}_{t_{k}}\left(x_{k},y\right)\right)}^{2}}{2{\;\overset{\leftarrow }{\;R\;}\;}_{t_{k}}\left(x_{k},y\right)}+o\left(1\right)\\ \; & \overset{\left(ii\right)}{≳}\min\left\{1,{\bar{\beta }}_{T-t_{k}}^{-1}\right\}\cdot E_{x_{t}∼{\;\overset{\leftarrow }{\;q\;}\;}_{t_{k}}}\sum_{y:y\neq x_{k}}{\left({\hat{R}}_{t_{k}}\left(x_{k},y\right)-{\;\overset{\leftarrow }{\;R\;}\;}_{t_{k}}\left(x_{k},y\right)\right)}^{2} \end{array}$$

where (i) follows by assuming that ${\hat{R}}_{t}\left(x,y\right)-{\;\overset{\leftarrow }{\;R\;}\;}_{t}\left(x,y\right)=o\left(1\right),\;\forall t,x,y$, and (ii) follows because $\frac{q_{t}\left(y\right)}{q_{t}\left(x_{k}\right)}\leq S\max\left\{1,{\bar{\beta }}_{t}^{-1}\right\}$ (see Lemma A2) and thus ${\;\overset{\leftarrow }{\;R\;}\;}_{t_{k}}\left(x_{k},y\right)\leq \max\left\{1,{\left(T-t_{k}\right)}^{-1}\right\}$. Therefore, we have that, for all $t_{k}$’s,

$$L_{TV}\left(s;T-t_{k}\right)≲\sqrt{d(S-1)}\max\left\{1,{\bar{\beta }}_{T-t_{k}}^{-1}\right\}\cdot \sqrt{L_{SE}\left(s;T-t_{k}\right)}.\;□$$

**Remark** **A1.**
*Here we have shown a point-wise upper bound over t. Indeed, a time-averaged upper bound can also be achieved with the step size in Theorem 2: $t_{k+1}-t_{k}=\kappa \min\left\{1,{\bar{\beta }}_{T-t_{k}}\right\}$, which yields*

$$\sum_{k=0}^{N-1}\left(t_{k+1}-t_{k}\right)L_{TV}\left(s;T-t_{k}\right)≲T\sqrt{dS}\underset{k\in [N]}{\sup}\sqrt{L_{SE}\left(s;T-t_{k}\right)}.$$

## Appendix B. Proof of Theorem 1

We follow the idea of [17,18] to analyze the Euler method by constructing a truncated version of the vanilla τ-leaping sampler. In [17] (Lemma 8), it is shown that the truncated τ-leaping sampler is asymptotically equivalent to the Euler method. Also, from Lemma 7 of [17], one important property of this sampler is that its sampling rate ${\hat{R}}_{t}$ is piecewise constant and, given $x_{t_{k}}\in {\left[S\right]}^{d}$, we have

(A1) $${\hat{R}}_{t}\left(x_{t_{k}},\cdot \right)={\hat{R}}_{t_{k}}\left(x_{t_{k}},\cdot \right).$$

Step 1: Decompose total error.

To begin, by [18] (Theorem 1), we have

$$\mathrm{TV}\left({\;\overset{\leftarrow }{\;q\;}\;}_{T-\delta },p_{T-\delta }\right)\leq \mathrm{TV}\left({\;\overset{\leftarrow }{\;q\;}\;}_{0},p_{0}\right)+\sum_{k=0}^{N-1}\int_{t_{k}}^{t_{k+1}}E_{x_{t}∼{\;\overset{\leftarrow }{\;q\;}\;}_{t}}\sum_{y:y\neq x_{t}}\left|{\hat{R}}_{t}\left(x_{t},y\right)-{\;\overset{\leftarrow }{\;R\;}\;}_{t}\left(x_{t},y\right)\right|dt.$$

Thus, if we write $h_{t}\left(x_{t}\right):=\sum_{y:y\neq x_{t}}\left|{\hat{R}}_{t}\left(x_{t},y\right)-{\;\overset{\leftarrow }{\;R\;}\;}_{t}\left(x_{t},y\right)\right|$, we have

(A2) $$\begin{array}{cc} \; & \mathrm{TV}({\;\overset{\leftarrow }{\;q\;}\;}_{T-\delta },p_{T-\delta })\\ \; & \leq \mathrm{TV}\left({\;\overset{\leftarrow }{\;q\;}\;}_{0},p_{0}\right)+\sum_{k=0}^{N-1}\int_{t_{k}}^{t_{k+1}}E_{x_{t}∼{\;\overset{\leftarrow }{\;q\;}\;}_{t}}\left[h_{t}\left(x_{t}\right)\right]dt\\ \; & =\mathrm{TV}\left({\;\overset{\leftarrow }{\;q\;}\;}_{0},p_{0}\right)+\sum_{k=0}^{N-1}\int_{t_{k}}^{t_{k+1}}E_{x_{t_{k}}∼{\;\overset{\leftarrow }{\;q\;}\;}_{t_{k}}}\left[h_{t_{k}}\left(x_{t_{k}}\right)\right]dt\\ \; & \;+\sum_{k=0}^{N-1}\int_{t_{k}}^{t_{k+1}}E_{\begin{array}{c} x_{t}∼{\;\overset{\leftarrow }{\;q\;}\;}_{t}\\ \;x_{t_{k}}∼{\;\overset{\leftarrow }{\;q\;}\;}_{t_{k}} \end{array}}\left[h_{t}\left(x_{t}\right)-h_{t_{k}}\left(x_{t_{k}}\right)\right]\\ \; & =\underset{\mathrm{initialization}\;\mathrm{error}}{\underbrace{\mathrm{TV}({\;\overset{\leftarrow }{\;q\;}\;}_{0},p_{0})}}+\underset{\mathrm{estimation}\;\mathrm{error}}{\underbrace{\sum_{k=0}^{N-1}\left(t_{k+1}-t_{k}\right)E_{x_{t_{k}}∼{\;\overset{\leftarrow }{\;q\;}\;}_{t_{k}}}\left[h_{t_{k}}\left(x_{t_{k}}\right)\right]}}\\ \; & \;+\underset{\mathrm{discretization}\;\mathrm{error}}{\underbrace{\sum_{k=0}^{N-1}\int_{t_{k}}^{t_{k+1}}E_{\begin{array}{c} x_{t}∼{\;\overset{\leftarrow }{\;q\;}\;}_{t}\\ x_{t_{k}}∼{\;\overset{\leftarrow }{\;q\;}\;}_{t_{k}} \end{array}}\left[h_{t}\left(x_{t}\right)-h_{t}\left(x_{t_{k}}\right)\right]+E_{x_{t_{k}}∼{\;\overset{\leftarrow }{\;q\;}\;}_{t_{k}}}\left[h_{t}\left(x_{t_{k}}\right)-h_{t_{k}}\left(x_{t_{k}}\right)\right]dt}}. \end{array}$$

By Assumption 1, the estimation error satisfies that

$$\sum_{k=0}^{N-1}\left(t_{k+1}-t_{k}\right)E_{x_{t_{k}}∼{\;\overset{\leftarrow }{\;q\;}\;}_{t_{k}}}\left[h_{t_{k}}\left(x_{t_{k}}\right)\right]\leq \sqrt{\varepsilon_{\mathrm{TV}}}.$$

Step 2: Bound initialization error.

The following lemma bounds the initialization error for a non-homogeneous noise schedule.

**Lemma** **A1.**
*Given the forward process in (1) with the rate given in (2), we have*

$$\mathrm{TV}\left(q_{T},p_{0}\right)≲\min\left\{d,\sqrt{dS}\right\}e^{-{\bar{\beta }}_{T}}.$$

**Proof.** See Appendix F.1. □

Step 3: Bound discretization error.

It now remains to upper-bound the discretization error. As shown in (A2), the discretization error can be decomposed into two terms, one for the time-difference in the argument of $h_{t}$ (in expected value) and the other in the space-difference for $h_{t}$ itself. Using [18] (Lemma 4), the space-difference term can be upper-bounded as

(A3) $$\begin{array}{cc} E_{\begin{array}{c} x_{t}∼{\;\overset{\leftarrow }{\;q\;}\;}_{t}\\ x_{t_{k}}∼{\;\overset{\leftarrow }{\;q\;}\;}_{t_{k}} \end{array}}\left[h_{t}\left(x_{t}\right)-h_{t}\left(x_{t_{k}}\right)\right] & \leq \left(t-t_{k}\right)E_{x_{t}∼{\;\overset{\leftarrow }{\;q\;}\;}_{t}}\left[\left(-R_{T-t}\left(x_{t},x_{t}\right)\right)h_{t}\left(x_{t}\right)\right]+o\left(t-t_{k}\right)\\ \; & ≲\left(t-t_{k}\right)d\cdot \beta_{T-t}E_{x_{t}∼{\;\overset{\leftarrow }{\;q\;}\;}_{t}}\left[h_{t}\left(x_{t}\right)\right]. \end{array}$$

Thus, since $t_{k+1}-t_{k}\leq \kappa$, we have

(A4) $$\begin{array}{cc} \; & \sum_{k=0}^{N-1}\int_{t_{k}}^{t_{k+1}}E_{\begin{array}{c} x_{t}∼{\;\overset{\leftarrow }{\;q\;}\;}_{t}\\ x_{t_{k}}∼{\;\overset{\leftarrow }{\;q\;}\;}_{t_{k}} \end{array}}\left[h_{t}\left(x_{t}\right)-h_{t}\left(x_{t_{k}}\right)\right]dt\\ \; & =\kappa \cdot d\sum_{k=0}^{N-1}\int_{t_{k}}^{t_{k+1}}\beta_{T-t}E_{x_{t}∼{\;\overset{\leftarrow }{\;q\;}\;}_{t}}\left[h_{t}\left(x_{t}\right)\right]dt\\ \; & \overset{\left(i\right)}{=}\kappa \cdot d\sum_{k=0}^{N-1}\int_{t_{k}}^{t_{k+1}}\frac{\beta_{T-t}}{1-\left(t-t_{k}\right)d\beta_{T-t}}E_{x_{t_{k}}∼{\;\overset{\leftarrow }{\;q\;}\;}_{t_{k}}}\left[h_{t}\left(x_{t_{k}}\right)\right]dt\\ \; & =\kappa \cdot d\sum_{k=0}^{N-1}\int_{t_{k}}^{t_{k+1}}\frac{\beta_{T-t}}{1-\left(t-t_{k}\right)d\beta_{T-t}}\\ \; & \;\cdot \left(E_{x_{t_{k}}∼{\;\overset{\leftarrow }{\;q\;}\;}_{t_{k}}}\left[h_{t_{k}}\left(x_{t_{k}}\right)\right]+E_{x_{t_{k}}∼{\;\overset{\leftarrow }{\;q\;}\;}_{t_{k}}}\left[h_{t}\left(x_{t_{k}}\right)-h_{t_{k}}\left(x_{t_{k}}\right)\right]\right)dt\\ \; & \overset{\left(ii\right)}{≲}\kappa \cdot d\underset{t}{\sup}\frac{\beta_{T-t}}{1-\left(t-t_{k}\right)d\beta_{T-t}}\left(\sqrt{\varepsilon }+\int_{0}^{T-\delta }E_{x_{t_{k}}∼{\;\overset{\leftarrow }{\;q\;}\;}_{t_{k}}}\left[h_{t}\left(x_{t_{k}}\right)-h_{t_{k}}\left(x_{t_{k}}\right)\right]dt\right) \end{array}$$

where (i) follows again by (A3), and (ii) follows by Assumption 1. Thus, as long as κ=o(1), the time-difference in the discretization error dominates.

We now turn to the time-difference term of the discretization error, which equals to

(A5) $$\begin{array}{cc} \; & E_{x_{t_{k}}∼{\;\overset{\leftarrow }{\;q\;}\;}_{t_{k}}}\left[h_{t}\left(x_{t_{k}}\right)-h_{t_{k}}\left(x_{t_{k}}\right)\right]\\ \; & =E_{x_{t_{k}}∼{\;\overset{\leftarrow }{\;q\;}\;}_{t_{k}}}\sum_{y:y\neq x_{t_{k}}}\left(|{\hat{R}}_{t}\left(x_{t_{k}},y\right)-{\;\overset{\leftarrow }{\;R\;}\;}_{t}\left(x_{t_{k}},y\right)\left|-\right|{\hat{R}}_{t_{k}}\left(x_{t_{k}},y\right)-{\;\overset{\leftarrow }{\;R\;}\;}_{t_{k}}\left(x_{t_{k}},y\right)|\right)\\ \; & \leq E_{x_{t_{k}}∼{\;\overset{\leftarrow }{\;q\;}\;}_{t_{k}}}\sum_{y:y\neq x_{t_{k}}}\left|{\hat{R}}_{t}\left(x_{t_{k}},y\right)-{\;\overset{\leftarrow }{\;R\;}\;}_{t}\left(x_{t_{k}},y\right)-{\hat{R}}_{t_{k}}\left(x_{t_{k}},y\right)+{\;\overset{\leftarrow }{\;R\;}\;}_{t_{k}}\left(x_{t_{k}},y\right)\right|\\ \; & \overset{\left(i\right)}{=}E_{x_{t_{k}}∼{\;\overset{\leftarrow }{\;q\;}\;}_{t_{k}}}\sum_{y:y\neq x_{t_{k}}}\left|{\hat{R}}_{t_{k}}\left(x_{t_{k}},y\right)-{\;\overset{\leftarrow }{\;R\;}\;}_{t}\left(x_{t_{k}},y\right)-{\hat{R}}_{t_{k}}\left(x_{t_{k}},y\right)+{\;\overset{\leftarrow }{\;R\;}\;}_{t_{k}}\left(x_{t_{k}},y\right)\right|\\ \; & =E_{x_{t_{k}}∼{\;\overset{\leftarrow }{\;q\;}\;}_{t_{k}}}\sum_{y:y\neq x_{t_{k}}}\left|{\;\overset{\leftarrow }{\;R\;}\;}_{t}\left(x_{t_{k}},y\right)-{\;\overset{\leftarrow }{\;R\;}\;}_{t_{k}}\left(x_{t_{k}},y\right)\right|, \end{array}$$

where (i) follows from the property of the sampler given in (A1).

Step 4: Bound Expected Absolute Difference in the Rate Function.

Continuing (A5), the rate-determining term is the expected absolute difference in the rate function. To this end, we need a characterization of the score function under a non-homogeneous noise schedule, as follows.

**Lemma** **A2** (Score bound under non-homogeneous $\beta_{t}$)**.** *Fix t>0 and x≠y such that $\mathrm{Ham}\left(x,y\right)=p$. Given the forward process in (1) with a rate given in (2), we have*

$$\frac{q_{t}\left(y\right)}{q_{t}\left(x\right)}≲{\left(S\cdot \max\{1,{\left({\bar{\beta }}_{t}\right)}^{-1}\}\right)}^{p}.$$

**Proof.** See Appendix F.2. □

Now, for any $x_{t_{k}}\in {\left[S\right]}^{d}$, the sum difference in the reverse rate can be further calculated using (3) as

(A6) $$\begin{array}{cc} \; & E_{x_{t_{k}}∼{\;\overset{\leftarrow }{\;q\;}\;}_{t_{k}}}\sum_{y\neq x_{t_{k}}}\left|{\;\overset{\leftarrow }{\;R\;}\;}_{t_{k}}\left(x_{t_{k}},y\right)-{\;\overset{\leftarrow }{\;R\;}\;}_{t}\left(x_{t_{k}},y\right)\right|\\ \; & =E_{x_{t_{k}}∼{\;\overset{\leftarrow }{\;q\;}\;}_{t_{k}}}\sum_{y\neq x_{t_{k}}}\left|\frac{q_{T-t_{k}}\left(y\right)}{q_{T-t_{k}}\left(x_{t_{k}}\right)}R_{T-t_{k}}\left(y,x_{t_{k}}\right)-\frac{q_{T-t}\left(y\right)}{q_{T-t}\left(x_{t_{k}}\right)}R_{T-t}\left(y,x_{t_{k}}\right)\right|\\ \; & \leq E_{x_{t_{k}}∼{\;\overset{\leftarrow }{\;q\;}\;}_{t_{k}}}\sum_{y\neq x_{t_{k}}}\left|\frac{q_{T-t_{k}}\left(y\right)}{q_{T-t_{k}}\left(x_{t_{k}}\right)}-\frac{q_{T-t}\left(y\right)}{q_{T-t}\left(x_{t_{k}}\right)}\right|R_{T-t_{k}}\left(y,x_{t_{k}}\right)\\ \; & \;+E_{x_{t_{k}}∼{\;\overset{\leftarrow }{\;q\;}\;}_{t_{k}}}\sum_{y\neq x_{t_{k}}}\frac{q_{T-t}\left(y\right)}{q_{T-t}\left(x_{t_{k}}\right)}\left|R_{T-t_{k}}\left(y,x_{t_{k}}\right)-R_{T-t}\left(y,x_{t_{k}}\right)\right|\\ \; & =E_{x_{t_{k}}∼{\;\overset{\leftarrow }{\;q\;}\;}_{t_{k}}}\sum_{\begin{array}{c} y\neq x_{t_{k}}\\ \mathrm{Ham}\left(y,x_{t_{k}}\right)=1 \end{array}}\left|\frac{q_{T-t_{k}}\left(y\right)}{q_{T-t_{k}}\left(x_{t_{k}}\right)}-\frac{q_{T-t}\left(y\right)}{q_{T-t}\left(x_{t_{k}}\right)}\right|R_{T-t_{k}}\left(y,x_{t_{k}}\right)\\ \; & \;+E_{x_{t_{k}}∼{\;\overset{\leftarrow }{\;q\;}\;}_{t_{k}}}\sum_{\begin{array}{c} y\neq x_{t_{k}}\\ \mathrm{Ham}\left(y,x_{t_{k}}\right)=1 \end{array}}\frac{q_{T-t}\left(y\right)}{q_{T-t}\left(x_{t_{k}}\right)}\left|R_{T-t_{k}}\left(y,x_{t_{k}}\right)-R_{T-t}\left(y,x_{t_{k}}\right)\right|, \end{array}$$

where the last line follows because $R_{t}\left(y,x\right)=0$ whenever $\mathrm{Ham}\left(y,x\right)\geq 2$. Here the second term captures the effect of non-homogeneity of $\beta_{t}$, which becomes zero in the case of constant $\beta_{t}$.

We first deal with the second term in (A6). Since $\beta_{t}$ is smooth, we have

$$|R_{T-t_{k}}\left(y,x_{t_{k}}\right)-R_{T-t}\left(y,x_{t_{k}}\right)|=\frac{1}{S}|\beta_{T-t_{k}}-\beta_{T-t}|≲\frac{1}{S}\left|\beta_{T-t_{k}}^{\prime }\right|\left(t-t_{k}\right).$$

Thus, combining the result from Lemma A2, the second term in (A6) satisfies that

$$\begin{array}{cc} \; & E_{x_{t_{k}}∼{\;\overset{\leftarrow }{\;q\;}\;}_{t_{k}}}\sum_{\begin{array}{c} y\neq x_{t_{k}}\\ \mathrm{Ham}\left(y,x_{t_{k}}\right)=1 \end{array}}\frac{q_{T-t}\left(y\right)}{q_{T-t}\left(x_{t_{k}}\right)}\left|R_{T-t_{k}}\left(y,x_{t_{k}}\right)-R_{T-t}\left(y,x_{t_{k}}\right)\right|\\ \; & ≲Sd|\beta_{T-t_{k}}^{\prime }|\max\left\{1,{\bar{\beta }}_{T-t_{k+1}}^{-1}\right\}\left(t-t_{k}\right). \end{array}$$

In order to provide an upper bound for the first term in (A6), it is essential to deal with the time difference of the likelihood ratios. The following lemma is a direct extension of [17] (Lemma 5).

**Lemma** **A3.** *Fix s<t such that t−s is small. Given the forward process in* (1) *with a rate given in* (2), *we have*

$$\begin{array}{cc} E_{x_{t}∼q_{t}}\sum_{\begin{array}{c} y\neq x_{t}\\ \mathrm{Ham}\left(y,x_{t}\right)=1 \end{array}}|\frac{q_{t}\left(y\right)}{q_{t}\left(x_{t}\right)}- & \frac{q_{s}\left(y\right)}{q_{s}\left(x_{t}\right)}|R_{t}\left(y,x_{t}\right)\\ & ≲dS\beta_{t}^{2}\max\left\{1,{\bar{\beta }}_{s}^{-2}\right\}\left(t-s\right)+d^{2}S\beta_{t}^{2}\max\left\{1,{\bar{\beta }}_{s}^{-1}\right\}\left(t-s\right). \end{array}$$

**Proof.** See Appendix F.3. □

Thus, considering the two terms in (A6), we have the following bound.

(A7) $$\begin{array}{cc} \; & E_{x_{t_{k}}∼{\;\overset{\leftarrow }{\;q\;}\;}_{t_{k}}}\sum_{y\neq x_{t_{k}}}\left|{\;\overset{\leftarrow }{\;R\;}\;}_{t_{k}}\left(x_{t_{k}},y\right)-{\;\overset{\leftarrow }{\;R\;}\;}_{t}\left(x_{t_{k}},y\right)\right|\\ \; & ≲dS\max\left\{1,{\bar{\beta }}_{T-t_{k+1}}^{-1}\right\}\left(\right|\beta_{T-t_{k}}^{\prime }\left|+\beta_{t}^{2}\max\left\{1,{\bar{\beta }}_{T-t_{k+1}}^{-1}\right\}+d\beta_{t}^{2}\right)\left(t-t_{k}\right). \end{array}$$

Therefore, combining all the ingredients above, we have

$$\begin{array}{cc} \; & \mathrm{TV}({\;\overset{\leftarrow }{\;q\;}\;}_{T-\delta },p_{T-\delta })\\ \; & ≲\min\left\{d,\sqrt{dS}\right\}e^{-{\bar{\beta }}_{T}}+\sqrt{\varepsilon_{\mathrm{TV}}}+dS\sum_{k=0}^{N-1}\max\left\{1,{\bar{\beta }}_{T-t_{k+1}}^{-2}\right\}\left(\right|\beta_{T-t_{k}}^{\prime }\left|+d\beta_{T-t_{k}}^{2}\right){\left(t-t_{k}\right)}^{2}. \end{array}$$

Step 5: Bound initial perturbation.

As we are finishing up the proof, it remains to investigate how much perturbation would be introduced due to early-stopping when $\beta_{t}$ is non-homogeneous. This is characterized in the following lemma, whose proof is similar to the last part of [12] (Theorem 6).

**Lemma** **A4.**
*Under the forward process in (1) with the rate given in (2), we have*

$$\mathrm{TV}\left(q_{0},q_{\delta }\right)≲d{\bar{\beta }}_{\delta },\;\mathrm{as}\;\delta \to 0.$$

**Proof.** See Appendix F.4. □

The proof of Theorem 1 is now complete.

## Appendix C. Proof of Theorem 2

From the result of Theorem 1, it remains to determine the number of steps using the special step sizes. The goal is to directly characterize the summation term, which we perform as follows.

Let $\mathrm{fn}\left(\beta_{T-t_{k}}\right)\in \{\beta_{T-t_{k}}^{2},|\beta_{T-t_{k}}^{\prime }\left|\right\}$. Recall that ${\bar{\beta }}_{t}$ is increasing. Define

$$k^{*}:=\sup\left\{k:{\bar{\beta }}_{T-t_{k}}>1\right\}.$$

We thus have the following cases.

1. Case 1: $k<k^{*}$. This implies that ${\bar{\beta }}_{T-t_{k}}>{\bar{\beta }}_{T-t_{k+1}}>1$, and $t_{k+1}-t_{k}\leq \kappa$. Thus, we have
  $$\sum_{k=0}^{k^{*}-1}{\left(t_{k+1}-t_{k}\right)}^{2}\frac{\mathrm{fn}(\beta_{T-t_{k}})}{\min\left\{1,{\bar{\beta }}_{T-t_{k+1}}^{2}\right\}}\leq \kappa^{2}\sum_{k=0}^{k^{*}-1}\mathrm{fn}\left(\beta_{T-t_{k}}\right).$$
2. Case 2: $k>k^{*}$. This implies that ${\bar{\beta }}_{T-t_{k+1}}<{\bar{\beta }}_{T-t_{k}}\leq 1$, and
  $$\begin{array}{cc} \sum_{k=k^{*}+1}^{N-1}{\left(t_{k+1}-t_{k}\right)}^{2}\frac{\mathrm{fn}(\beta_{T-t_{k}})}{\min\left\{1,{\bar{\beta }}_{T-t_{k+1}}^{2}\right\}} & \leq \kappa^{2}\sum_{k=k^{*}+1}^{N-1}\mathrm{fn}\left(\beta_{T-t_{k}}\right)\frac{{\bar{\beta }}_{T-t_{k}}^{2}}{{\bar{\beta }}_{T-t_{k+1}}^{2}}\\ \; & ≍\kappa^{2}\sum_{k=k^{*}+1}^{N-1}\mathrm{fn}\left(\beta_{T-t_{k}}\right) \end{array}$$
  where the last line follows because by the Taylor expansion of $\bar{\beta }$ (noting that $\bar{\beta }$ is continuous since by definition it is the integral of $\beta_{t}$),
  (A8) $${\bar{\beta }}_{T-t_{k}}={\bar{\beta }}_{T-t_{k+1}}+O\left(t_{k+1}-t_{k}\right)={\bar{\beta }}_{T-t_{k+1}}+O\left(\kappa \right),\;\forall k=0,\ldots ,N-1.$$
  Since ${\bar{\beta }}_{T-t_{k+1}}\geq {\bar{\beta }}_{\delta }>0$, we have ${\bar{\beta }}_{T-t_{k}}^{2}/{\bar{\beta }}_{T-t_{k+1}}^{2}=1+O\left(\kappa \right)=1+o\left(1\right)$ for all k=0,…,N−1.
3. Case 3: $k=k^{*}$. This implies that ${\bar{\beta }}_{T-t_{k+1}}\leq 1$, ${\bar{\beta }}_{T-t_{k}}>1$, and $t_{k+1}-t_{k}\leq \kappa$. Also, from (A8), ${\bar{\beta }}_{T-t_{k}}\leq 1+O\left(\kappa \right)$. Then,
  $${\left(t_{k+1}-t_{k}\right)}^{2}\frac{\mathrm{fn}(\beta_{T-t_{k}})}{\min\left\{1,{\bar{\beta }}_{T-t_{k+1}}^{2}\right\}}≍\kappa^{2}\mathrm{fn}\left(\beta_{T-t_{k}}\right)\frac{{\bar{\beta }}_{T-t_{k}}^{2}}{{\bar{\beta }}_{T-t_{k+1}}^{2}}≍\kappa^{2}\mathrm{fn}\left(\beta_{T-t_{k^{*}}}\right).$$

Summing up all three cases, we have

$$\sum_{k=0}^{N-1}{\left(t_{k+1}-t_{k}\right)}^{2}\frac{\mathrm{fn}(\beta_{T-t_{k}})}{\min\left\{1,{\bar{\beta }}_{T-t_{k+1}}^{2}\right\}}≲\kappa^{2}\sum_{k=0}^{N-1}\mathrm{fn}\left(\beta_{T-t_{k}}\right).$$

Now, under Assumption 2, since $\beta_{t}$ and $|\beta_{t}^{\prime }|$ have upper bounds independent of the system parameters, we have

$$\sum_{k=0}^{N-1}{\left(t_{k+1}-t_{k}\right)}^{2}\frac{\mathrm{fn}(\beta_{T-t_{k}})}{\min\left\{1,{\bar{\beta }}_{T-t_{k+1}}^{2}\right\}}≲\kappa^{2}N.$$

Next, we choose $t_{k+1}-t_{k}\leq \kappa \min\left\{1,{\bar{\beta }}_{T-t_{k}}\right\}$ and we need to determine an upper bound for the number of steps. Define $t^{*}=t_{k^{*}}$. When $k\leq k^{*}$, the number of steps is upper- bounded as

$$k^{*}=:N_{1}=\left\lfloor \frac{T-t^{*}}{\kappa }\right\rfloor \leq \frac{T}{\kappa }.$$

When $k>k^{*}$, note that $t_{k+1}$ is chosen according to the following fixed-point iteration: $T-t_{k+1}=h\left(T-t_{k}\right)$, where $h\left(z\right):=z-\kappa {\bar{\beta }}_{z}$. Obviously z=0 is a fixed point. By Banach’s fixed-point theorem,

$$T-t_{N_{2}}\leq \left(T-t^{*}\right)\cdot {\left(\max_{z\in [\delta ,T-t^{*}]}\left|h^{\prime }\left(z\right)\right|\right)}^{N_{2}}.$$

Here note that $h^{\prime }\left(z\right)=1-\kappa \beta_{z}\leq 1-\kappa \beta^{*}$, where we recall that $\beta^{*}=\min_{z\in [\delta ,{\bar{\beta }}^{-1}\left(1\right)]}\beta_{z}$. Thus, in order to reach that $T-t_{N_{2}}≍\delta$,

$$N_{2}≲\log_{1-\kappa \beta^{*}}\frac{\delta }{T-t^{*}}≍\frac{\log\left(T-t^{*}\right)+\log\delta^{-1}}{\kappa \beta^{*}}.$$

Therefore, the total number of steps to take satisfies that

$$N=N_{1}+N_{2}≲\frac{T+\left(\log T+\log\delta^{-1}\right)/\beta^{*}}{\kappa }.$$

Finally, since under Assumption 2 we have $\beta^{*}≳1$, this yields

$$N≲\frac{T+\log\delta^{-1}}{\kappa }.$$

Then, we have

$$\sum_{k=0}^{N-1}{\left(t_{k+1}-t_{k}\right)}^{2}\frac{\mathrm{fn}(\beta_{T-t_{k}})}{\min\left\{1,{\bar{\beta }}_{T-t_{k+1}}^{2}\right\}}≲\kappa \left(T+\log\delta^{-1}\right).$$

Note that $T≲\mathrm{poly}(\log\left(d/\sqrt{\varepsilon }\right))$. Choosing the corresponding *T* and κ yields the desired result.

## Appendix D. Proof of Theorem 3

We begin from Theorem 1, which also applies to the constant step size:

$$\begin{array}{cc} \mathrm{TV}\left(q_{0},p_{T-\delta }\right)≲\min\{d & ,\sqrt{dS}\}e^{-{\bar{\beta }}_{T}}+\sqrt{\varepsilon_{\mathrm{TV}}}\\ & +dS\sum_{k=0}^{N-1}\max\left\{1,{\bar{\beta }}_{T-t_{k+1}}^{-2}\right\}\left(\right|\beta_{T-t_{k}}^{\prime }\left|+d\beta_{T-t_{k}}^{2}\right){\left(t_{k+1}-t_{k}\right)}^{2}+d{\bar{\beta }}_{\delta }. \end{array}$$

We recall from (7) that

$${\bar{\beta }}_{t}:=\beta_{\min}^{1-t}\beta_{\max}^{t},\;\beta_{t}=\beta_{\min}^{1-t}\beta_{\max}^{t}\log\left(\beta_{\max}/\beta_{\min}\right),\;\beta_{t}^{\prime }=\beta_{\min}^{1-t}\beta_{\max}^{t}\log^{2}\left(\beta_{\max}/\beta_{\min}\right).$$

Thus, we have ${\bar{\beta }}_{T}=\beta_{\max}$. Also, note that

$${\bar{\beta }}_{\delta }=\beta_{\min}^{1-\delta }\beta_{\max}^{\delta }\overset{\delta \to 0}{\to }\beta_{\min}>0,$$

which implies that there will be asymptotic mismatch for vanishing δ. What remains is to work out the summation term, and we proceed as follows.

Note that

$$\begin{array}{cc} \; & \sum_{k=0}^{N-1}\kappa \max\left\{1,{\bar{\beta }}_{T-t_{k+1}}^{-2}\right\}\left(\right|\beta_{T-t_{k}}^{\prime }\left|+d\beta_{T-t_{k}}^{2}\right)\\ \; & =\log^{2}\left(\beta_{\max}/\beta_{\min}\right)\sum_{k=0}^{N-1}\kappa \max\left\{1,\beta_{\min}^{-2t_{k+1}}\beta_{\max}^{-2(1-t_{k+1})}\right\}\left(\beta_{\min}^{t_{k}}\beta_{\max}^{1-t_{k}}+d\beta_{\min}^{2t_{k}}\beta_{\max}^{2(1-t_{k})}\right)\\ \; & ≍\log^{2}\left(\beta_{\max}/\beta_{\min}\right)\int_{\delta }^{1}\max\left\{1,\beta_{\min}^{-2(1-t)}\beta_{\max}^{-2t}\right\}\left(\beta_{\min}^{1-t}\beta_{\max}^{t}+d\beta_{\min}^{2(1-t)}\beta_{\max}^{2t}\right)dt. \end{array}$$

Since $\beta_{\min}<1<\beta_{\max}$, and ${\bar{\beta }}_{t}$ is smooth, there exists $t^{*}$ such that ${\bar{\beta }}_{t^{*}}=1$. Then, the integral becomes

$$\begin{array}{cc} \; & \log\left(\beta_{\max}/\beta_{\min}\right)\int_{\delta }^{1}\max\left\{1,\beta_{\min}^{-2(1-t)}\beta_{\max}^{-2t}\right\}\left(\beta_{\min}^{1-t}\beta_{\max}^{t}+d\beta_{\min}^{2(1-t)}\beta_{\max}^{2t}\right)dt\\ \; & =\log\left(\beta_{\max}/\beta_{\min}\right)\int_{\delta }^{t^{*}}\beta_{\min}^{-2(1-t)}\beta_{\max}^{-2t}\left(\beta_{\min}^{1-t}\beta_{\max}^{t}+d\right)dt+\int_{t^{*}}^{1}\beta_{\min}^{1-t}\beta_{\max}^{t}+d\beta_{\min}^{2(1-t)}\beta_{\max}^{2t}dt\\ \; & =-{\left.\frac{1}{{\bar{\beta }}_{t}}\right|}_{\delta }^{t^{*}}-{\left.\frac{d}{2{\bar{\beta }}_{t}^{2}}\right|}_{\delta }^{t^{*}}+{\left.{\bar{\beta }}_{t}\right|}_{t^{*}}^{1}+\frac{d}{2}{\left.{\bar{\beta }}_{t}^{2}\right|}_{t^{*}}^{1}\\ \; & ≍\beta_{\min}^{-1}-1+\frac{d}{2}{\left(\beta_{\min}^{-1}-1\right)}^{2}+\left(\beta_{\max}-1\right)+\frac{d}{2}{\left(\beta_{\max}-1\right)}^{2}\\ \; & ≲d\left({\left(\beta_{\min}^{-1}-1\right)}^{2}+{\left(\beta_{\max}-1\right)}^{2}\right). \end{array}$$

Combining all of the above, we finally have, for each δ>0,

$$\begin{array}{cc} \mathrm{TV}\left(q_{0},p_{T-\delta }\right)≲\min\{d, & \sqrt{dS}\}e^{-\beta_{\max}}+\sqrt{\varepsilon_{\mathrm{TV}}}\\ & +\kappa d^{2}S\log\left(\beta_{\max}/\beta_{\min}\right)\left({\left(\beta_{\min}^{-1}-1\right)}^{2}+{\left(\beta_{\max}-1\right)}^{2}\right)+d\beta_{\min}. \end{array}$$

Since the right-hand side does not depend on δ, taking its limit to $0^{+}$ yields the desired result.

## Appendix E. Proof of Theorem 4

Continuing the proof of Theorem 3, we again have

$$\begin{array}{cc} \; & \sum_{k=0}^{N-1}{\left(t_{k+1}-t_{k}\right)}^{2}\max\left\{1,{\bar{\beta }}_{T-t_{k+1}}^{-2}\right\}\left(\right|\beta_{T-t_{k}}^{\prime }\left|+d\beta_{T-t_{k}}^{2}\right)\\ \; & =\log^{2}\left(\beta_{\max}/\beta_{\min}\right)\sum_{k=0}^{N-1}{\left(t_{k+1}-t_{k}\right)}^{2}\max\left\{1,{\bar{\beta }}_{T-t_{k+1}}^{-2}\right\}\left(\beta_{\min}^{t_{k}}\beta_{\max}^{1-t_{k}}+d\beta_{\min}^{2t_{k}}\beta_{\max}^{2(1-t_{k})}\right) \end{array}$$

Now, suppose we choose step sizes as in Theorem 2:

(A9) $$t_{k+1}-t_{k}=\kappa \min\left\{1,{\bar{\beta }}_{T-t_{k}}\right\}=\kappa \min\left\{1,\beta_{\min}^{t_{k}}\beta_{\max}^{1-t_{k}}\right\}.$$

Again define

$$k^{*}:=\sup\left\{k:{\bar{\beta }}_{T-t_{k}}=\beta_{\min}^{t_{k}}\beta_{\max}^{1-t_{k}}>1\right\},$$

with $t^{*}$ such that $\beta_{\min}^{t^{*}}\beta_{\max}^{1-t^{*}}=1$. Then, from the proof of Theorem 2, using this step size, we have

$$\begin{array}{cc} \; & \log^{2}\left(\beta_{\max}/\beta_{\min}\right)\sum_{k=0}^{N-1}{\left(t_{k+1}-t_{k}\right)}^{2}\max\left\{1,{\bar{\beta }}_{T-t_{k+1}}^{-2}\right\}\left(\beta_{\min}^{t_{k}}\beta_{\max}^{1-t_{k}}+d\beta_{\min}^{2t_{k}}\beta_{\max}^{2(1-t_{k})}\right)\\ \; & ≲\log^{2}\left(\beta_{\max}/\beta_{\min}\right)\kappa^{2}\sum_{k=0}^{N-1}\left(\beta_{\min}^{t_{k}}\beta_{\max}^{1-t_{k}}+d\beta_{\min}^{2t_{k}}\beta_{\max}^{2(1-t_{k})}\right) \end{array}$$

For $k<k^{*}$, $t_{k+1}-t_{k}=\kappa$. This is the regime similar to Theorem 3, where we have

$$\begin{array}{cc} \; & \sum_{k=0}^{k^{*}-1}\left(\beta_{\min}^{t_{k}}\beta_{\max}^{1-t_{k}}+d\beta_{\min}^{2t_{k}}\beta_{\max}^{2(1-t_{k})}\right)\\ \; & ≍\frac{1}{\kappa }\int_{t^{*}}^{1}\beta_{\min}^{1-t}\beta_{\max}^{t}+d\beta_{\min}^{2(1-t)}\beta_{\max}^{2t}dt\\ \; & =\frac{1}{\kappa \log(\beta_{\max}/\beta_{\min})}\left(\left(\beta_{\max}-1\right)+\frac{d}{2}{\left(\beta_{\max}-1\right)}^{2}\right). \end{array}$$

Meanwhile, the number of steps in this regime, $N_{1}$, satisfies that

$$N_{1}≍\frac{1-t^{*}}{\kappa }\leq \frac{1}{\kappa }.$$

As follows, the goal is thus to provide an upper bound on the two summation terms as well as $N_{2}$ in the regime where $k\geq k^{*}$. The key idea is to use the property that ${\bar{\beta }}_{t}$, $\beta_{t}$, and $\beta_{t}^{\prime }$ have the same exponential form in *t*.

Part 1: On the number of steps N 2.

Let us define a series of auxiliary variable $y_{k}$ as follows. Let

$$y_{k}:={\bar{\beta }}_{1-t_{k}}^{-1}={\left(\beta_{\min}^{t_{k}}\beta_{\max}^{1-t_{k}}\right)}^{-1}.$$

Here, by definition of $k^{*}$, we have $y_{k^{*}}≍1$. Also notice that $y_{k}$ is increasing in *k*, with

$$\frac{y_{k+1}}{y_{k}}=\beta_{\min}^{-(t_{k+1}-t_{k})}\beta_{\max}^{t_{k+1}-t_{k}}=e^{\lambda (t_{k+1}-t_{k})},$$

where $\lambda :=\log(\beta_{\max}/\beta_{\min})$. With the step size in (A9) and since $k\geq k^{*}$, we have

$$\frac{y_{k+1}}{y_{k}}=e^{\lambda \kappa \beta_{\min}^{t_{k}}\beta_{\max}^{1-t_{k}}}=e^{\frac{\lambda \kappa }{y_{k}}}\geq 1+\frac{\lambda \kappa }{y_{k}},$$

which further implies that

$$y_{k+1}\geq y_{k}+\lambda \kappa .$$

For the terminal condition, we have $y_{N}≍{\left(\beta_{\min}^{1-\delta }\beta_{\max}^{\delta }\right)}^{-1}$. This implies that

$$N_{2}=N-k^{*}≲\frac{{\left(\beta_{\min}^{1-\delta }\beta_{\max}^{\delta }\right)}^{-1}-1}{\lambda \kappa }\overset{\delta \to 0}{\to }\frac{\beta_{\min}^{-1}-1}{\lambda \kappa }.$$

Part 2: On the first-order sum.

With (A9) and when $k\geq k^{*}$, the first-order sum is indeed a telescoping sum:

$$\sum_{k=k^{*}}^{N-1}\beta_{\min}^{t_{k}}\beta_{\max}^{1-t_{k}}=\sum_{k=k^{*}}^{N-1}\frac{t_{k+1}-t_{k}}{\kappa }=\frac{1}{\kappa }\left(t_{N}-t_{k^{*}}\right)\leq \frac{1-\delta }{\kappa }.$$

Part 3: On the second-order sum.

The second-order sum needs some extra work. The idea is to upper-bound it with the first-order sum using trajectory smoothness. Write

(A10) $$f_{k}:=y_{k}^{-1}=\beta_{\min}^{t_{k}}\beta_{\max}^{1-t_{k}}.$$

Note that $f_{k}\leq 1$ for $k>k^{*}$ (for $k=k^{*}$, we get $f_{k}=1+o\left(1\right)$), and we have a similar recursion as the above one:

$$f_{k+1}=f_{k}e^{-\lambda \kappa f_{k}}.$$

The following lemma characterizes an important property of $f_{k}$, which will be useful for the analysis.

**Lemma** **A5.**
*With $f_{k}$ defined in (A10) and when $k>k^{*}$, we have*

$$f_{k}^{2}\leq \frac{f_{k}-f_{k+1}}{1-e^{-\lambda \kappa }},$$

*where $\lambda :=\log(\beta_{\max}/\beta_{\min})$.*

**Proof.** See Appendix G. □

With Lemma A5, the second-order sum becomes

$$\begin{array}{cc} d\sum_{k=k^{*}}^{N-1}\beta_{\min}^{2t_{k}}\beta_{\max}^{2(1-t_{k})} & =d\sum_{k=k^{*}}^{N-1}f_{k}^{2}\\ \; & ≲d\sum_{k=k^{*}}^{N-1}\frac{f_{k}-f_{k+1}}{1-e^{-\lambda \kappa }}\\ \; & =\frac{d}{1-e^{-\lambda \kappa }}\left(t_{N}-t_{k^{*}}\right)\\ \; & ≲\frac{d}{\lambda \kappa }\left(1-\delta \right). \end{array}$$

Part 4: Combine all previous parts.

The overall discretization error becomes

$$\begin{array}{cc} \; & \log^{2}\left(\beta_{\max}/\beta_{\min}\right)\kappa^{2}\sum_{k=0}^{N-1}\left(\beta_{\min}^{t_{k}}\beta_{\max}^{1-t_{k}}+d\beta_{\min}^{2t_{k}}\beta_{\max}^{2(1-t_{k})}\right)\\ \; & ≲\log^{2}\left(\beta_{\max}/\beta_{\min}\right)\kappa^{2}\frac{1}{\kappa \log(\beta_{\max}/\beta_{\min})}\left(\left(\beta_{\max}-1\right)+\frac{d}{2}{\left(\beta_{\max}-1\right)}^{2}\right)\\ \; & \;+\log^{2}\left(\beta_{\max}/\beta_{\min}\right)\kappa^{2}\left(\frac{1-\delta }{\kappa }+\frac{d}{\log(\beta_{\max}/\beta_{\min})\kappa }\left(1-\delta \right)\right)\\ \; & ≲d\log\left(\beta_{\max}/\beta_{\min}\right)\kappa \left({\left(\beta_{\max}-1\right)}^{2}+1-\delta \right)\\ \; & \overset{\delta \to 0}{\to }d\log\left(\beta_{\max}/\beta_{\min}\right)\kappa \left({\left(\beta_{\max}-1\right)}^{2}+1\right). \end{array}$$

Also note that the total number of steps satisfies

$$N=N_{1}+N_{2}≲\frac{1}{\kappa }\left(1+\frac{\beta_{\min}^{-1}-1}{\log(\beta_{\max}/\beta_{\min})}\right).$$

The proof is now complete.

## Appendix F. Auxiliary Proofs

In this section, we provide proofs of all the auxiliary lemmas in this paper.

### Appendix F.1. Proof of Lemma A1

Write $\epsilon_{T}:=e^{-{\bar{\beta }}_{T}}$. Let $u_{S}$ denote the uniform distribution on [S], and let $u_{d}=u_{S}^{⊗d}$. By assumption, $p_{0}=u_{d}$.

First, to obtain the analytical solution for the conditional probability, we can solve the Kolmogorov forward equation for the *i*-th dimension (∀i∈[d]):

$$\frac{d}{dt}q_{t|0}^{i}\left(z|a\right)=\sum_{\tilde{z}\in \left[S\right]}q_{t|0}^{i}\left(\tilde{z}|a\right)R_{t}^{\mathrm{tok}}\left(\tilde{z},z\right),$$

whose solution is

(A11) $$\begin{array}{cc} q_{t|0}^{i}\left(z|a\right) & =\left[\exp\left(\int_{0}^{t}R_{s}^{\mathrm{tok}}ds\right)\right]\left(a,z\right)\\ \; & =\left[\exp\left({\bar{\beta }}_{t}R_{\mathrm{base}}\right)\right]\left(a,z\right)\\ \; & =\left[P\exp\left({\bar{\beta }}_{t}\Lambda \right)P^{-1}\right]\left(a,z\right)\\ \; & =\left\{\begin{array}{cc} S^{-1}\left(1-e^{-{\bar{\beta }}_{t}}\right) & \mathrm{if}\;z\neq a\\ S^{-1}\left(1+\left(S-1\right)e^{-{\bar{\beta }}_{t}}\right) & \mathrm{if}\;z=a \end{array}\right. \end{array}$$

where we recall that $R_{t}^{\mathrm{tok}}=\beta_{t}R_{\mathrm{base}}$ and we denote the eigendecomposition of $R_{\mathrm{base}}=S^{-1}1_{S}1_{S}^{⊺}-I_{S}$ as $R_{\mathrm{base}}=P\Lambda P^{-1}$. Equivalently, we can write

$$q_{T|0}^{i}\left(z|a\right)=u_{S}\left(z\right)+\epsilon_{T}\left(1\left\{z=a\right\}-u_{S}\left(z\right)\right).$$

Thus, for fixed $x_{0},x_{T}\in {\left[S\right]}^{d}$,

$$q_{T|0}\left(x_{T}|x_{0}\right)=\overset{d}{\underset{i=1}{⨂}}q_{T|0}^{i}\left(x_{T}^{i}|x_{0}^{i}\right).$$

To upper-bound the initialization error, we first reduce to the case of a fixed initialization. Since

$$q_{T}\left(x_{T}\right)=\sum_{x_{0}\in {\left[S\right]}^{d}}q_{0}\left(x_{0}\right)q_{T|0}\left(x_{T}|x_{0}\right),$$

by Jensen’s inequality we have

$$\mathrm{TV}\left(q_{T},p_{0}\right)\leq \sum_{x_{0}\in {\left[S\right]}^{d}}q_{0}\left(x_{0}\right)\mathrm{TV}\left(q_{T|0}\left(\cdot |x_{0}\right),p_{0}\right).$$

Therefore it suffices to upper-bound $\mathrm{TV}(q_{T|0}\left(\cdot |x_{0}\right),u_{d})$ uniformly over $x_{0}$.

For one coordinate, we have

$$\begin{array}{cc} \mathrm{TV}(q_{T|0}^{i}\left(\cdot |a\right),u_{S})\; & =\frac{1}{2}\sum_{z\in [S]}\left|q_{T|0}^{i}\left(z|a\right)-S^{-1}\right|\\ \; & =\frac{1}{2}\cdot \frac{S-1}{S}\epsilon_{T}+\frac{1}{2}\cdot \left(S-1\right)\frac{1}{S}\epsilon_{T}\\ \; & =\frac{S-1}{S}\epsilon_{T}. \end{array}$$

Using the standard tensorization bound for total variation of product measures,

$$\mathrm{TV}\left(\overset{d}{\underset{i=1}{⨂}}\mu_{i},\overset{d}{\underset{i=1}{⨂}}\nu_{i}\right)\leq 1-\prod_{i=1}^{d}\left(1-\mathrm{TV}(\mu_{i},\nu_{i})\right),$$

we obtain

$$\mathrm{TV}\left(q_{T|0}\left(\cdot |x_{0}\right),u_{d}\right)\leq 1-{\left(1-\frac{S-1}{S}\epsilon_{T}\right)}^{d}.$$

Consequently,

$$\mathrm{TV}\left(q_{T},p_{0}\right)\leq 1-{\left(1-\frac{S-1}{S}\epsilon_{T}\right)}^{d}\leq d\frac{S-1}{S}\epsilon_{T}\leq de^{-{\bar{\beta }}_{T}}.$$

We can also derive a complementary bound using the likelihood ratio. For fixed $x_{0}$, define

$$L_{x_{0}}\left(x_{T}\right):=\frac{q_{T|0}\left(x_{T}|x_{0}\right)}{u_{d}\left(x_{T}\right)}.$$

For one coordinate,

$$\frac{q_{T|0}^{i}\left(z|a\right)}{u_{S}\left(z\right)}=1+\epsilon_{T}\left(S1\{z=a\}-1\right).$$

Hence,

$$L_{x_{0}}\left(x_{T}\right)=\prod_{i=1}^{d}\left[1+\epsilon_{T}\left(S1\{x_{T}^{i}=x_{0}^{i}\}-1\right)\right].$$

By Cauchy–Schwarz,

$$\mathrm{TV}\left(q_{T|0}\left(\cdot |x_{0}\right),u_{d}\right)=\frac{1}{2}E_{x_{T}∼u_{d}}\left[|L_{x_{0}}\left(x_{T}\right)-1|\right]\leq \frac{1}{2}\sqrt{E_{x_{T}∼u_{d}}\left[{\left(L_{x_{0}}\left(x_{T}\right)-1\right)}^{2}\right]}.$$

Since $x_{T}^{1},\ldots ,x_{T}^{d}$ are independent under $u_{d}$,

$$\begin{array}{cc} E_{x_{T}∼u_{d}}\left[L_{x_{0}}{\left(x_{T}\right)}^{2}\right]\; & =\prod_{i=1}^{d}E_{z∼u_{S}}\left[{\left(1+\epsilon_{T}\left(S1\left\{z=x_{0}^{i}\right\}-1\right)\right)}^{2}\right]\\ \; & ={\left[\frac{1}{S}{\left(1+\left(S-1\right)\epsilon_{T}\right)}^{2}+\frac{S-1}{S}{\left(1-\epsilon_{T}\right)}^{2}\right]}^{d}\\ \; & ={\left(1+\left(S-1\right)\epsilon_{T}^{2}\right)}^{d}. \end{array}$$

Also, $E_{u_{d}}\left[L_{x_{0}}\right]=1$. Therefore,

$$E_{u_{d}}\left[{\left(L_{x_{0}}-1\right)}^{2}\right]={\left(1+\left(S-1\right)\epsilon_{T}^{2}\right)}^{d}-1,$$

and thus

$$\mathrm{TV}\left(q_{T|0}\left(\cdot |x_{0}\right),u_{d}\right)\leq \frac{1}{2}\sqrt{{\left(1+\left(S-1\right)\epsilon_{T}^{2}\right)}^{d}-1}.$$

Again using convexity over $x_{0}∼q_{0}$, this gives

$$\mathrm{TV}\left(q_{T},p_{0}\right)\leq \frac{1}{2}\sqrt{{\left(1+\left(S-1\right)\epsilon_{T}^{2}\right)}^{d}-1}.$$

Combining the two bounds yields

$$\mathrm{TV}\left(q_{T},p_{0}\right)\leq \left[1-{\left(1-\frac{S-1}{S}\epsilon_{T}\right)}^{d}\right]\wedge \frac{1}{2}\sqrt{{\left(1+\left(S-1\right)\epsilon_{T}^{2}\right)}^{d}-1}.$$

Finally, since $1-{\left(1-r\right)}^{d}\leq dr$ and ${\left(1+a\right)}^{d}\leq e^{ad}$, we obtain

$$\mathrm{TV}\left(q_{T},p_{0}\right)≲\min\left\{d\epsilon_{T},\;\sqrt{dS}\epsilon_{T}\right\}=\min\left\{d,\sqrt{dS}\right\}\epsilon_{T}.$$

### Appendix F.2. Proof of Lemma A2

Write $P:=\left\{i\in \left[d\right]:y^{i}\neq x^{i}\right\}$. First, we note that

(A12) $$\begin{array}{cc} \frac{q_{t}\left(y\right)}{q_{t}\left(x\right)} & =\frac{1}{q_{t}\left(x\right)}\sum_{x_{0}\in {\left[S\right]}^{d}}q_{0}\left(x_{0}\right)q_{t|0}\left(y|x_{0}\right)\\ \; & \overset{\left(i\right)}{=}\frac{1}{q_{t}\left(x\right)}\sum_{x_{0}\in {\left[S\right]}^{d}}q_{0}\left(x_{0}\right)\prod_{i\in [d]}q_{t|0}^{i}\left(y^{i}|x_{0}^{i}\right)\\ \; & =\frac{1}{q_{t}\left(x\right)}\sum_{x_{0}\in {\left[S\right]}^{d}}q_{0}\left(x_{0}\right)\left(\prod_{i\in [d]}q_{t|0}^{i}\left(x^{i}|x_{0}^{i}\right)\right)\left(\prod_{i\in P}\frac{q_{t|0}^{i}\left(x^{i}|x_{0}^{i}\right)}{q_{t|0}^{i}\left(y^{i}|x_{0}^{i}\right)}\right)\\ \; & \overset{\left(ii\right)}{=}\sum_{x_{0}\in {\left[S\right]}^{d}}\frac{q_{0}\left(x_{0}\right)q_{t|0}\left(x|x_{0}\right)}{q_{t}\left(x\right)}\left(\prod_{i\in P}\frac{q_{t|0}^{i}\left(x^{i}|x_{0}^{i}\right)}{q_{t|0}^{i}\left(y^{i}|x_{0}^{i}\right)}\right)\\ \; & =E_{x_{0}∼q_{0|t}\left(\cdot |x\right)}\prod_{i\in P}\frac{q_{t|0}^{i}\left(y^{i}|x_{0}^{i}\right)}{q_{t|0}^{i}\left(x^{i}|x_{0}^{i}\right)} \end{array}$$

where both (i) and (ii) follow because with the chosen $R_{t}$ in (2), each dimension propagates independently in the forward process (cf. [11] (Prop. 3)). Recall the forward conditional probability under non-homogeneity in (A11), which yields

(A13) $$\frac{q_{t|0}^{i}\left(y^{i}|x_{0}^{i}\right)}{q_{t|0}^{i}\left(x^{i}|x_{0}^{i}\right)}=\left\{\begin{array}{cc} 1 & \mathrm{if}\;x^{i}\neq x_{0}^{i}\;\mathrm{and}\;y^{i}\neq x_{0}^{i}\\ \frac{1-e^{-{\bar{\beta }}_{t}}}{1+\left(S-1\right)e^{-{\bar{\beta }}_{t}}} & \mathrm{if}\;x^{i}=x_{0}^{i}\;\mathrm{but}\;y^{i}\neq x_{0}^{i}\\ \frac{1+\left(S-1\right)e^{-{\bar{\beta }}_{t}}}{1-e^{-{\bar{\beta }}_{t}}} & \mathrm{if}\;x^{i}\neq x_{0}^{i}\;\mathrm{but}\;y^{i}=x_{0}^{i} \end{array}\right..$$

Now, since $e^{-{\bar{\beta }}_{t}}\geq 0$, the second case satisfies that $\frac{1-e^{-{\bar{\beta }}_{t}}}{1+\left(S-1\right)e^{-{\bar{\beta }}_{t}}}\leq 1$. For the third case, we have

$$\begin{array}{cc} \frac{1+\left(S-1\right)e^{-{\bar{\beta }}_{t}}}{1-e^{-{\bar{\beta }}_{t}}} & =1+S\cdot \frac{1}{e^{{\bar{\beta }}_{t}}-1}\leq \left\{\begin{array}{cc} S+1 & \mathrm{if}\;e^{{\bar{\beta }}_{t}}\geq 2\\ \frac{S}{{\bar{\beta }}_{t}} & \mathrm{otherwise} \end{array}\right.\\ \; & ≲S\cdot \max\{1,{\left({\bar{\beta }}_{t}\right)}^{-1}\}. \end{array}$$

Since these bounds do not depend on *i*, the proof is now complete.

### Appendix F.3. Proof of Lemma A3

First, fix $x_{t}$ and *y* and let *i* be the index such that $x_{t}^{i}\neq y^{i}$. From (A12), we have,

(A14) $$\begin{array}{cc} |\frac{q_{t}\left(y\right)}{q_{t}\left(x_{t}\right)}-\frac{q_{s}\left(y\right)}{q_{s}\left(x_{t}\right)}| & =|E_{x_{0}∼q_{0|t}\left(\cdot |x_{t}\right)}\left[\frac{q_{t|0}^{i}\left(y^{i}|x_{0}^{i}\right)}{q_{t|0}^{i}\left(x_{t}^{i}|x_{0}^{i}\right)}\right]-E_{{\tilde{x}}_{0}∼q_{0|s}\left(\cdot |x_{t}\right)}\left[\frac{q_{s|0}^{i}\left(y^{i}|{\tilde{x}}_{0}^{i}\right)}{q_{s|0}^{i}\left(x_{t}^{i}|{\tilde{x}}_{0}^{i}\right)}\right]|\\ \; & \leq E_{x_{0}∼q_{0|t}\left(\cdot |x_{t}\right)}\left|\frac{q_{t|0}^{i}\left(y^{i}|x_{0}^{i}\right)}{q_{t|0}^{i}\left(x_{t}^{i}|x_{0}^{i}\right)}-\frac{q_{s|0}^{i}\left(y^{i}|x_{0}^{i}\right)}{q_{s|0}^{i}\left(x_{t}^{i}|x_{0}^{i}\right)}\right|\\ \; & \;+|E_{\begin{array}{c} x_{0}∼q_{0|t}\left(\cdot |x_{t}\right)\\ {\tilde{x}}_{0}∼q_{0|s}\left(\cdot |x_{t}\right) \end{array}}\left[\frac{q_{s|0}^{i}\left(y^{i}|x_{0}^{i}\right)}{q_{s|0}^{i}\left(x_{t}^{i}|x_{0}^{i}\right)}-\frac{q_{s|0}^{i}\left(y^{i}|{\tilde{x}}_{0}^{i}\right)}{q_{s|0}^{i}\left(x_{t}^{i}|{\tilde{x}}_{0}^{i}\right)}\right]|. \end{array}$$

For the first term in (A14), we note the expression of likelihood ratio in (A13), and thus for any fixed $x_{0}$, $x_{t}$, and *y*,

$$|\frac{q_{t|0}^{i}\left(y^{i}|x_{0}^{i}\right)}{q_{t|0}^{i}\left(x_{t}^{i}|x_{0}^{i}\right)}-\frac{q_{s|0}^{i}\left(y^{i}|x_{0}^{i}\right)}{q_{s|0}^{i}\left(x_{t}^{i}|x_{0}^{i}\right)}|=\left\{\begin{array}{cc} 0 & \mathrm{if}\;x^{i}\neq x_{0}^{i}\;\mathrm{and}\;y^{i}\neq x_{0}^{i}\\ \frac{1-e^{-{\bar{\beta }}_{t}}}{1+\left(S-1\right)e^{-{\bar{\beta }}_{t}}}-\frac{1-e^{-{\bar{\beta }}_{s}}}{1+\left(S-1\right)e^{-{\bar{\beta }}_{s}}} & \mathrm{if}\;x^{i}=x_{0}^{i}\;\mathrm{but}\;y^{i}\neq x_{0}^{i}\\ \frac{1+\left(S-1\right)e^{-{\bar{\beta }}_{t}}}{1-e^{-{\bar{\beta }}_{t}}}-\frac{1+\left(S-1\right)e^{-{\bar{\beta }}_{s}}}{1-e^{-{\bar{\beta }}_{s}}} & \mathrm{if}\;x^{i}\neq x_{0}^{i}\;\mathrm{but}\;y^{i}=x_{0}^{i} \end{array}\right..$$

Now, since

$$\begin{array}{cc} |\frac{\partial }{\partial t}\left(\frac{1-e^{-{\bar{\beta }}_{t}}}{1+\left(S-1\right)e^{-{\bar{\beta }}_{t}}}\right)| & =\frac{Se^{{\bar{\beta }}_{t}}\beta_{t}}{{\left(S+e^{{\bar{\beta }}_{t}}-1\right)}^{2}}≲\beta_{t}\\ |\frac{\partial }{\partial t}\left(\frac{1+\left(S-1\right)e^{-{\bar{\beta }}_{t}}}{1-e^{-{\bar{\beta }}_{t}}}\right)| & =\frac{Se^{{\bar{\beta }}_{t}}\beta_{t}}{{\left(e^{{\bar{\beta }}_{t}}-1\right)}^{2}}≲\frac{S\beta_{t}}{\min{\left\{1,{\bar{\beta }}_{t}\right\}}^{2}}, \end{array}$$

we have

$$|\frac{q_{t|0}^{i}\left(y^{i}|x_{0}^{i}\right)}{q_{t|0}^{i}\left(x_{t}^{i}|x_{0}^{i}\right)}-\frac{q_{s|0}^{i}\left(y^{i}|x_{0}^{i}\right)}{q_{s|0}^{i}\left(x_{t}^{i}|x_{0}^{i}\right)}|≲\frac{S\beta_{t}}{\min{\left\{1,{\bar{\beta }}_{t}\right\}}^{2}}\left(t-s\right).$$

Note that this term does not depend on *d*. Thus,

$$\begin{array}{cc} E_{x_{t}∼q_{t}}\sum_{\begin{array}{c} y\neq x_{t}\\ \mathrm{Ham}\left(y,x_{t}\right)=1 \end{array}}E_{x_{0}∼q_{0|t}\left(\cdot |x_{t}\right)}\left|\frac{q_{t|0}^{i}\left(y^{i}|x_{0}^{i}\right)}{q_{t|0}^{i}\left(x_{t}^{i}|x_{0}^{i}\right)}-\frac{q_{s|0}^{i}\left(y^{i}|x_{0}^{i}\right)}{q_{s|0}^{i}\left(x_{t}^{i}|x_{0}^{i}\right)}\right|R_{t} & \left(y,x_{t}\right)\\ & ≲dS\beta_{t}^{2}\max\left\{1,{\bar{\beta }}_{t}^{-2}\right\}\left(t-s\right). \end{array}$$

Now we turn to the second term in (A14). Write $f\left(z\right):=\frac{q_{s|0}^{i}\left(y^{i}|z\right)}{q_{s|0}^{i}\left(x_{t}^{i}|z\right)}$ for brevity (recall that $x_{t}$ and *y* are fixed and thus omitted in this expression). Note that from (A13), an upper bound on f(z) is

$$\underset{y^{i},x_{t}^{i},z\in \left[S\right]}{\sup}f\left(z\right)=\underset{y^{i},x_{t}^{i},z\in \left[S\right]}{\sup}\frac{q_{s|0}^{i}\left(y^{i}|z\right)}{q_{s|0}^{i}\left(x_{t}^{i}|z\right)}≲S\cdot \max\left\{1,{\bar{\beta }}_{s}^{-1}\right\}.$$

Thus, the second term in (A14) can be upper-bounded (for each $y^{i}$) as

(A15) $$\begin{array}{cc} |E_{\begin{array}{c} x_{0}∼q_{0|t}\left(\cdot |x_{t}\right)\\ {\tilde{x}}_{0}∼q_{0|s}\left(\cdot |x_{t}\right) \end{array}}\left[f\left(x_{0}^{i}\right)-f\left({\tilde{x}}_{0}^{i}\right)\right]| & =|\sum_{x_{0}\in {\left[S\right]}^{d}}f\left(x_{0}^{i}\right)\left(q_{0|t}\left(x_{0}|x_{t}\right)-q_{0|s}\left(x_{0}|x_{t}\right)\right)|\\ \; & ≲S\max\left\{1,{\bar{\beta }}_{s}^{-1}\right\}\sum_{x_{0}\in {\left[S\right]}^{d}}\left|q_{0|t}\left(x_{0}|x_{t}\right)-q_{0|s}\left(x_{0}|x_{t}\right)\right|. \end{array}$$

Using Bayes’s rule, we have

(A16) $$\begin{array}{cc} \; & \sum_{x_{0}\in {\left[S\right]}^{d}}\left|q_{0|t}\left(x_{0}|x_{t}\right)-q_{0|s}\left(x_{0}|x_{t}\right)\right|\\ \; & =\sum_{x_{0}\in {\left[S\right]}^{d}}q_{0}\left(x_{0}\right)\left|\frac{q_{t|0}\left(x_{t}|x_{0}\right)}{q_{t}\left(x_{t}\right)}-\frac{q_{s|0}\left(x_{t}|x_{0}\right)}{q_{s}\left(x_{t}\right)}\right|\\ \; & \leq \frac{1}{q_{t}\left(x_{t}\right)\cdot q_{s}\left(x_{t}\right)}\sum_{x_{0},y_{0}\in {\left[S\right]}^{d}}q_{0}\left(x_{0}\right)q_{0}\left(y_{0}\right)\\ \; & \;\cdot |q_{t|0}\left(x_{t}|x_{0}\right)q_{s|0}\left(x_{t}|y_{0}\right)-q_{s|0}\left(x_{t}|x_{0}\right)q_{t|0}\left(x_{t}|y_{0}\right)|\\ \; & \leq \frac{1}{q_{t}\left(x_{t}\right)\cdot q_{s}\left(x_{t}\right)}E_{x_{0},y_{0}∼q_{0}}[\left|q_{t|0}\left(x_{t}|x_{0}\right)-q_{s|0}\left(x_{t}|x_{0}\right)\right|q_{s|0}\left(x_{t}|y_{0}\right)\\ \; & \;+|q_{s|0}\left(x_{t}|y_{0}\right)-q_{t|0}\left(x_{t}|y_{0}\right)|q_{s|0}\left(x_{t}|x_{0}\right)]\\ \; & =\frac{1}{q_{t}\left(x_{t}\right)}E_{x_{0}∼q_{0}}\left|q_{t|0}\left(x_{t}|x_{0}\right)-q_{s|0}\left(x_{t}|x_{0}\right)\right|\\ \; & \;+\frac{1}{q_{t}\left(x_{t}\right)}E_{y_{0}∼q_{0}}\left|q_{s|0}\left(x_{t}|y_{0}\right)-q_{t|0}\left(x_{t}|y_{0}\right)\right|\\ \; & =\frac{2}{q_{t}\left(x_{t}\right)}E_{x_{0}∼q_{0}}\left|q_{t|0}\left(x_{t}|x_{0}\right)-q_{s|0}\left(x_{t}|x_{0}\right)\right| \end{array}$$

Now, this term (without the constant factor 2) can be upper-bounded as

(A17) $$\begin{array}{cc} \; & \frac{1}{q_{t}\left(x_{t}\right)}E_{x_{0}∼q_{0}}\left|q_{t|0}\left(x_{t}|x_{0}\right)-q_{s|0}\left(x_{t}|x_{0}\right)\right|\\ \; & ≲\frac{1}{q_{t}\left(x_{t}\right)}\left(t-s\right)E_{x_{0}∼q_{0}}\left|\frac{\partial }{\partial t}q_{t|0}\left(x_{t}|x_{0}\right)\right|\\ \; & \overset{\left(i\right)}{=}\frac{1}{q_{t}\left(x_{t}\right)}\left(t-s\right)E_{x_{0}∼q_{0}}\left|\sum_{{\tilde{x}}_{t}\in {\left[S\right]}^{d}}q_{t|0}\left({\tilde{x}}_{t}|x_{0}\right)R_{t}\left({\tilde{x}}_{t},x_{t}\right)\right|\\ \; & \leq \frac{1}{q_{t}\left(x_{t}\right)}\left(t-s\right)E_{x_{0}∼q_{0}}\sum_{{\tilde{x}}_{t}\in {\left[S\right]}^{d}}q_{t|0}\left({\tilde{x}}_{t}|x_{0}\right)\left|R_{t}\left({\tilde{x}}_{t},x_{t}\right)\right|\\ \; & =\left(t-s\right)\sum_{{\tilde{x}}_{t}\in {\left[S\right]}^{d}}\frac{q_{t}\left({\tilde{x}}_{t}\right)}{q_{t}\left(x_{t}\right)}\left|R_{t}\left({\tilde{x}}_{t},x_{t}\right)\right|, \end{array}$$

where (i) follows from Kolmogorov forward equation. Thus, combining these intermediate results, we have

$$\begin{array}{cc} \; & E_{x_{t}∼q_{t}}\sum_{\begin{array}{c} y\neq x_{t}\\ \mathrm{Ham}\left(y,x_{t}\right)=1 \end{array}}\left|E_{\begin{array}{c} x_{0}∼q_{0|t}\left(\cdot |x_{t}\right)\\ {\tilde{x}}_{0}∼q_{0|s}\left(\cdot |x_{t}\right) \end{array}}\left[f\left(x_{0}^{i}\right)-f\left({\tilde{x}}_{0}^{i}\right)\right]\right|R_{t}\left(y,x_{t}\right)\\ \; & \overset{\left(ii\right)}{≲}dS\beta_{t}\max\left\{1,{\bar{\beta }}_{s}^{-1}\right\}\cdot E_{x_{t}∼q_{t}}\left[\frac{1}{q_{t}\left(x_{t}\right)}E_{x_{0}∼q_{0}}\left|q_{t|0}\left(x_{t}|x_{0}\right)-q_{s|0}\left(x_{t}|x_{0}\right)\right|\right]\\ \; & \overset{\left(iii\right)}{≲}\left(t-s\right)dS\beta_{t}\max\left\{1,{\bar{\beta }}_{s}^{-1}\right\}E_{x_{t}∼q_{t}}\left[\sum_{{\tilde{x}}_{t}\in {\left[S\right]}^{d}}\frac{q_{t}\left({\tilde{x}}_{t}\right)}{q_{t}\left(x_{t}\right)}\left|R_{t}\left({\tilde{x}}_{t},x_{t}\right)\right|\right]\\ \; & =\left(t-s\right)dS\beta_{t}\max\left\{1,{\bar{\beta }}_{s}^{-1}\right\}E_{{\tilde{x}}_{t}∼q_{t}}\sum_{x_{t}\in {\left[S\right]}^{d}}\left|R_{t}\left({\tilde{x}}_{t},x_{t}\right)\right|\\ \; & \overset{\left(iv\right)}{≍}\left(t-s\right)d^{2}S\beta_{t}^{2}\max\left\{1,{\bar{\beta }}_{s}^{-1}\right\}, \end{array}$$

where (ii) follows from (A15) and (A16), (iii) follows from (A17), and (iv) follows because $-R_{t}\left(x,x\right)=\sum_{y\neq x}R_{t}\left(x,y\right)=\beta_{t}\frac{S-1}{S}d$ for all $x,y\in {\left[S\right]}^{d}$. The proof is now complete.

### Appendix F.4. Proof of Lemma A4

Write $\Pi (q_{0},q_{\delta })$ is the set of all joint probability measures with marginal distributions $q_{0}$ and $q_{\delta }$. Then,

$$\begin{array}{cc} \mathrm{TV}(q_{0},q_{\delta }) & =\min_{\pi \in \Pi (q_{0},q_{\delta })}E_{x_{0},x_{\delta }∼\pi }1\left\{x_{\delta }\neq x_{0}\right\}\\ \; & \leq E_{x_{0}∼q_{0}}\left[Q_{\delta |0}\left\{x_{\delta }\neq x_{0}\right\}\right]\\ \; & \overset{\left(i\right)}{\leq }\sum_{i\in [d]}\sum_{a\neq x_{0}^{i}}E_{x_{0}∼q_{0}}\left(Q_{\delta |0}^{i}\left\{a\neq x_{0}^{i}\right\}\right)\\ \; & \overset{\left(ii\right)}{=}d\frac{S-1}{S}\left(1-e^{-{\bar{\beta }}_{\delta }}\right)\\ \; & ≍d{\bar{\beta }}_{\delta }, \end{array}$$

where (i) follows from the union bound, and (ii) follows from (A11). The proof is now complete.

## Appendix G. Proof of Lemma A5

Recall the recursion of $f_{k}$ as

$$f_{k+1}=f_{k}e^{-\lambda \kappa f_{k}}.$$

Equivalently, we have

$$\frac{f_{k}-f_{k+1}}{1-e^{-\lambda \kappa }}=\frac{f_{k}\left(1-e^{-\lambda \kappa f_{k}}\right)}{1-e^{-\lambda \kappa }}.$$

With this, since $f_{k}>0$, it is equivalent to prove that

$$f_{k}\leq \frac{1-e^{-\lambda \kappa f_{k}}}{1-e^{-\lambda \kappa }}\Leftrightarrow 1-e^{-\lambda \kappa }\leq \frac{1-e^{-\lambda \kappa f_{k}}}{f_{k}}.$$

Now, since the function $g\left(z\right)=\frac{1-e^{-az}}{z}$ is decreasing in z>0 for a>0, we get the latter inequality by noting that $f_{k}\leq 1$ for $k>k^{*}$.
